# Descriptor-guided thermodynamic screening of $\hbox {H}_2$ adsorption on single-atom-doped anatase $\hbox {TiO}_2$ nanoparticles with interpretable machine learning

**DOI:** 10.1038/s41598-026-55149-w

**Published:** 2026-05-27

**Authors:** Mustafa Kurban, Can Polat, Erchin Serpedin, Hasan Kurban

**Affiliations:** 1 Department of Prosthetics and Orthotics, Ankara University, Ankara, Turkey; 2 Department of Electrical and Computer Engineering, Texas A&M University at Qatar, Doha, Qatar; 3 Department of Electrical and Computer Engineering, Texas A&M University, College Station, TX USA; 4 College of Science and Engineering, Hamad Bin Khalifa University, Doha, Qatar

**Keywords:** $\hbox {H}_2$ adsorption, Anatase $\hbox {TiO}_2$ nanoparticles, Single-atom doping, Thermodynamic screening, Interpretable machine learning, Chemistry, Materials science, Nanoscience and technology

## Abstract

Understanding how surface dopants tune $\hbox {H}_2$ adsorption on oxide nanoparticles is important for the design of reversible hydrogen-storage materials and catalytic interfaces. Here, we present a descriptor-guided screening study of molecular $\hbox {H}_2$ adsorption on pristine and single-atom-doped anatase $\hbox {TiO}_2$ nanoparticles using density-functional tight-binding calculations, conceptual DFT descriptors, thermodynamic modelling, and interpretable machine learning. The replacement of one surface Ti atom with Al, Fe, Hf, La, Mo, Nb, Sn, V, W, or Zr enables systematic comparison across chemically distinct adsorption environments. Most dopants preserve molecular adsorption, whereas Fe shows incipient dissociative activation, and the adsorption energies span from *-0.275* to *-0.523* eV, indicating that single-atom doping can tune $\hbox {H}_2$ binding over a practically relevant range. Descriptor analysis separates weakly perturbed wide-gap systems from narrow-gap dopants with dopant-derived frontier states, enhanced softness, and higher electrophilicity. Symbolic regression with leave-one-out cross-validation identifies a compact $\omega ^{-}$-dependent expression (the electron-donating power, a member of the electrophilicity descriptor family) as the most generalizing descriptor–property relationship in this small-data regime, with higher-complexity formulas exhibiting an overfitting cliff; the large in-sample sensitivity to charge-transfer descriptors in those higher-complexity formulas reflects the chosen symbolic form rather than a directly measurable adsorption-energy variation. Gaussian-process modelling is retained as an uncertainty-driven active-learning sampling-design tool. Thermodynamic screening further shows that Nb and Zr provide the most balanced uptake–release behavior, Sn remains borderline viable, and Hf and Mo define a stronger-binding but less balanced regime. Overall, the workflow provides a data-efficient and physically interpretable basis for screening dopant chemistry in oxide nanomaterials.

## Introduction

Hydrogen is widely regarded as a key energy carrier for decarbonizing hard-to-electrify sectors, yet its widespread deployment continues to be limited by the lack of storage technologies that are simultaneously safe, compact, and reversible under practical operating conditions^1–4^. Conventional storage routes based on compressed gas or cryogenic liquid hydrogen impose substantial energetic and infrastructural penalties, which has sustained strong interest in solid-state storage concepts. Among these, adsorption-based storage is particularly attractive because it avoids bulk hydride formation and can, in principle, enable rapid and reversible uptake/release cycles. Its practical viability, however, depends on a narrow thermodynamic balance: $\hbox {H}_2$ must bind strongly enough to provide measurable uptake, yet weakly enough to be released within realistic pressure–temperature swings near ambient conditions^1,3^. Local coordination engineering, including metal decoration and Kubas-type binding motifs, therefore remains a central strategy for tuning adsorption strength beyond pure physisorption without sacrificing reversibility^5^.

Within this context, $\hbox {TiO}_2$ is an especially attractive oxide platform. It is abundant, chemically robust, structurally versatile, and supported by an exceptionally rich surface-science literature, making it a useful model system for linking local electronic structure to adsorption behaviour^6^. Anatase $\hbox {TiO}_2$ nanoparticles are particularly appealing because their high surface-to-volume ratio, low-coordination surface sites, and tunable facet exposure provide a broad spectrum of chemically distinct adsorption environments^7^. Equally important, the chemistry of anatase can be modified in a controlled way through substitutional doping, heteroatom incorporation, and defect engineering, allowing systematic tuning of local structure, charge distribution, and adsorption-site reactivity^8,9^. Hydrogen–$\hbox {TiO}_2$ interactions are known to be highly sensitive to this local environment: on stoichiometric surfaces molecular adsorption is typically weak, whereas oxygen vacancies, reduced Ti sites, and surface modifiers can substantially alter adsorption geometry, activation barriers, and charge transfer^10–13^. Beyond its fundamental importance, $\hbox {TiO}_2$ has also shown practical promise as a tunable hydrogen-adsorbing support, where targeted decoration or doping can enhance uptake through cooperative electronic and spillover-like effects^14–16^.

The central challenge is to convert these largely system-specific insights into predictive, design-oriented rules that generalize across dopant chemistries. This challenge has motivated growing interest in data-driven materials modelling, where descriptor-based learning can help bridge the gap between quantum-chemical calculations and materials screening^17–22^. However, adsorption studies on doped oxide nanoparticles typically operate in a small-data regime: each new candidate requires an expensive electronic-structure calculation, while the accessible design space is far larger than what can be exhaustively sampled. In such settings, black-box models are often statistically fragile and physically opaque, whereas interpretable and uncertainty-aware methods are far better matched to the scale of the problem^23^. Symbolic regression (SR) is attractive because it yields compact closed-form relationships between descriptors and target properties, thereby supporting direct physical interpretation^24^. Gaussian process (GP) regression complements this by providing calibrated posterior uncertainty from very limited data, making it a natural basis for active learning in scarce-data materials design^25^. Conceptual DFT (CDFT) descriptors, such as chemical potential, hardness, softness, and electrophilicity, are especially valuable in this setting because they encode intrinsic electronic reactivity in a compact and physically meaningful feature space^26,27^.

In this work, we develop a descriptor-guided screening framework for $\hbox {H}_2$ adsorption on pristine and single-atom-doped anatase $\hbox {TiO}_2$ nanoparticles, using the Hydrogent framework to integrate quantum-chemical modelling, thermodynamic analysis, and interpretable machine learning within a single workflow. By systematically probing a chemically diverse dopant space, we show that the hydrogen-storage behaviour of $\hbox {TiO}_2$ nanoparticles can be rationalized in terms of compact electronic descriptors that link local dopant chemistry to adsorption strength, reactivity, and deliverability. This shifts the problem from case-by-case energetic comparison toward physically grounded surrogate design in the small-data regime. The combination of CDFT descriptors, symbolic regression, Gaussian-process uncertainty quantification, and prospective candidate ranking on unseen dopants shows that physically interpretable screening rules can be extracted from a limited number of electronic-structure calculations. More broadly, the framework introduced here provides a general route for interpretable and data-efficient dopant optimization in oxide nanomaterials for hydrogen storage.

The remainder of the paper is organized as follows. Section Methodology describes the nanoparticle models, electronic-structure calculations, descriptor construction, thermodynamic analysis, and machine-learning workflow. Section Results and Discussion presents the adsorption energetics, descriptor trends, surrogate-model performance, active-learning behaviour, and forward-screening results. Section Conclusions summarizes the main findings and outlines future directions.

## Methodology

### Nanoparticle construction and surface doping

Anatase $\hbox {TiO}_2$ nanoparticle models were constructed using a cut-from-bulk approach. The starting structure was generated from a large anatase $\hbox {TiO}_2$ bulk supercell with dimensions of $60 \times 60 \times 60$, from which a nearly spherical cluster was excised by applying a radial cutoff centred within the supercell. The resulting pristine nanoparticle has an approximate diameter of 1.8 nm and contains 269 atoms ($\hbox {Ti}_{{89}}$ $\hbox {O}_{{180}}$), thereby providing a finite model with a diverse range of low-coordination surface environments relevant to adsorption.

To examine the influence of dopant chemistry on hydrogen adsorption, a series of single-atom-doped $\hbox {TiO}_2$ nanoparticles was constructed by substitutionally replacing one surface Ti atom with a dopant species *X*, where *X* = Al, Fe, Hf, La, Mo, Nb, Sn, V, W, and Zr. Surface substitution was chosen in order to probe adsorption-active local environments while preserving direct accessibility of the dopant site to the adsorbing $\hbox {H}_2$ molecule. The selected dopants span a chemically diverse set of main-group and transition-metal elements with distinct valence configurations, ionic radii, and electronic characters, thereby allowing a systematic assessment of dopant-dependent adsorption trends.

For consistency, the same substitutional motif was used for all doped models. This controlled substitutional motif should be interpreted as a surface-accessible dopant model for comparing dopant chemistry, rather than as a claim that the selected site is the global thermodynamic ground state for every dopant under all synthesis conditions. The selected dopants have literature precedent for incorporation into, or electronic/structural modification of, anatase $\hbox {TiO}_2$ systems; for example, Zr-doped $\hbox {TiO}_2$ nanoparticles can retain the anatase nanostructure and show improved thermal stability, Sn-doped $\hbox {TiO}_2$ can remain phase-pure anatase, and Nb-doped $\hbox {TiO}_2$ is widely discussed as a substitutionally doped anatase system with modified conduction-band/electron-transport behavior^28–30^. A complete dopant-stability and segregation analysis would require formation-energy comparisons across surface, subsurface, and core sites under synthesis-dependent chemical potentials. The present work instead addresses the adsorption-screening question for surface-accessible dopant motifs: how dopant identity modifies molecular $\hbox {H}_2$ adsorption when the local substitution environment is held fixed across the series. After model construction, both pristine and doped nanoparticles were fully relaxed prior to adsorption calculations to obtain well-defined reference structures and to ensure that the computed adsorption energetics reflect the intrinsic response of each nanoparticle to local dopant incorporation.

### Electronic structure calculations

All electronic-structure calculations were performed within the density-functional tight-binding (DFTB) framework using the DFTB+ code^31^. The Slater–Koster interactions were described using the PTBP parameter set^32^, a broadly transferable solid-state DFTB parametrization derived against PBE-level DFT reference data through the simultaneous optimization of the electronic and repulsive contributions across chemically diverse elemental crystal prototypes. Given its reported transferability to multicomponent solid-state systems, including oxides, PTBP provides a computationally tractable and internally consistent Hamiltonian for comparative screening of relative adsorption trends across the present dopant series. In this sense, it is used here as a screening-level baseline rather than as a one-to-one replacement for higher-level DFT adsorption energetics. To account for long-range dispersion effects, which are important for a consistent description of weak $\hbox {H}_2$–surface interactions, the DFT-D3 correction with Becke–Johnson damping was included throughout^33,34^.

Geometry optimizations were carried out using the rational optimizer as implemented in DFTB+. A maximum of 3000 optimization steps was allowed, and convergence thresholds of 0.02 eV $\hbox {AA}^{-1}$ for the maximum force component and $10^{-5}$ eV for the total energy were applied. Electronic occupations were treated using Fermi filling at 300 K.

For each nanoparticle model, including the pristine and all single-atom-doped anatase $\hbox {TiO}_2$ nanoparticles, both the pristine and the $\hbox {H}_2$-adsorbed configuration were structurally relaxed prior to analysis. To maintain the overall nanoparticle framework while still allowing local structural adaptation at the surface, geometry relaxation was restricted to the adsorption-relevant surface region, consistent with previous nanocluster adsorption studies showing that the dominant relaxation effects are typically localized around the adsorbate and the near-surface atoms, whereas more highly coordinated core atoms can be kept fixed with limited loss of accuracy^35^. For each system, $\hbox {H}_2$ adsorption was benchmarked from multiple physically distinct initial placements, including approximately perpendicular and parallel approaches as well as additional trial orientations at different angular dispositions and starting distances relative to the local adsorption region. The resulting adsorption energies are therefore interpreted as motif-specific values for a controlled surface-substitution environment rather than as arbitrary single-site estimates. This controlled-site strategy isolates the chemical role of the dopant by keeping the local substitution geometry fixed across the full dopant series, so that differences in adsorption energy can be assigned primarily to dopant identity rather than to changes in surface coordination or facet type. Within each fixed dopant motif, multiple $\hbox {H}_2$ starting orientations and distances were relaxed, and the lowest-energy converged adsorption geometry was retained. This provides a consistent and internally comparable basis for descriptor construction, thermodynamic screening, and dopant ranking across the present series. The resulting converged structures were used in all subsequent energetic, thermodynamic, and electronic analyses.

### $\hbox {H}_2$ Adsorption energetics and structural descriptors

The adsorption energy of a single $\hbox {H}_2$ molecule on the nanoparticle surface was calculated as

1 $$\begin{aligned} E_{\textrm{ads}} = E_{\mathrm {NP+H_2}} - E_{\textrm{NP}} - E_{\mathrm {H_2}}, \end{aligned}$$

where $E_{\mathrm {NP+H_2}}$, $E_{\textrm{NP}}$, and $E_{\mathrm {H_2}}$ denote the total energies of the optimized adsorbed system, the relaxed pristine nanoparticle, and an isolated $\hbox {H}_2$ molecule, respectively. Negative values of $E_{\textrm{ads}}$ correspond to exothermic adsorption.

To characterize the local adsorption geometry, structural descriptors were extracted from the relaxed configurations. In particular, the H–H bond length was used to monitor the degree of molecular activation upon adsorption, while the distance between the $\hbox {H}_2$ molecule and the nearest surface metal centre (Ti or dopant site) was used to quantify the adsorption configuration. These descriptors provide a compact structural measure of adsorption strength and incipient $\hbox {H}_2$ activation across chemically distinct surface environments.

### Conceptual DFT descriptors

The electronic reactivity of each nanoparticle configuration was characterized using a set of twelve CDFT descriptors evaluated at the DFTB level. These descriptors were chosen to provide a compact yet chemically interpretable representation of the electronic structure relevant to $\hbox {H}_2$ adsorption. In addition to the frontier orbital energies, namely the HOMO ($E_\textrm{H}$) and LUMO ($E_\textrm{L}$), we considered the ionization potential (IP) and electron affinity (EA), from which the standard global reactivity indices were constructed. The chemical potential was defined as $\mu = -(\textrm{IP} + \textrm{EA})/2$, the global hardness as $\eta = \textrm{IP} - \textrm{EA}$, the global softness as *S = 1/(2η )*, the electrophilicity index as $\omega = \mu ^2/(2\eta )$, and the electronegativity as $\chi = -\mu$. To further resolve donor–acceptor tendencies, we also included the electron-donating power, $\omega ^- = (3\textrm{IP}+\textrm{EA})^2/(16\eta )$, the electron-accepting power, $\omega ^+ = (\textrm{IP}+3\textrm{EA})^2/(16\eta )$, and the maximum fractional electron transfer, $\Delta N_{\max } = -\mu /\eta$. Taken together, these descriptors capture complementary aspects of electronic stability, charge-transfer propensity, and electrophilic or nucleophilic character, and were therefore used as the primary descriptor space for adsorption analysis and machine-learning model construction^26,27^.

Because several of these quantities are algebraically related, a degree of multicollinearity is intrinsic to the descriptor set. Rather than eliminating such dependencies *a priori*, we retained the full descriptor space and addressed redundancy during downstream analysis through symbolic regression, which performs implicit feature selection, and correlation-based importance ranking.

### Thermodynamic screening

Adsorption energy alone is not sufficient to evaluate the practical relevance of a candidate for hydrogen storage; the operating pressure–temperature window must also be taken into account^36^. To assess the thermodynamic viability of the pristine and doped $\hbox {TiO}_2$ nanoparticles under application-relevant conditions, we therefore supplemented the static adsorption-energy analysis with a Langmuir-type equilibrium model and van’t Hoff analysis.

Assuming single-site adsorption, the fractional surface coverage *θ* at pressure *P* and temperature *T* was described using the Langmuir isotherm^37^,

2 $$\begin{aligned} \theta (P, T) = \frac{K(T)\,P}{1 + K(T)\,P}, \end{aligned}$$

where *K*(*T*) is the equilibrium constant for adsorption. In the present model, *K*(*T*) was expressed as

3 $$\begin{aligned} K(T) = K_0 \exp (-E_{\textrm{ads}}/k_B T), \end{aligned}$$

where $E_{\textrm{ads}}$ is expressed on a per-molecule basis and is therefore paired with $k_{\textrm{B}}T$. In the present calculations, $E_{\textrm{ads}}$ is reported in eV per $\hbox {H}_2$ molecule and converted consistently when used in thermodynamic expressions. The prefactor $K_0$ was estimated from the standard molar entropy of $\hbox {H}_2$ after applying the corresponding molecular-to-molar unit conversion. This treatment enables a direct comparison of the expected temperature- and pressure-dependent uptake behaviour across the nanoparticle series.

To further quantify adsorption thermodynamics, the temperature dependence of the equilibrium constant was analyzed using the van’t Hoff relation,

4 $$\begin{aligned} \ln K = -\frac{\Delta H}{RT} + \frac{\Delta S}{R}, \end{aligned}$$

from which the effective enthalpic and entropic contributions to adsorption were estimated. Here, *Δ H* and *Δ S* are molar thermodynamic quantities and *R* is the molar gas constant. Thus, Eq. (3) and Eq. (4) use consistent energy conventions: $k_{\textrm{B}}T$ is used for the per-molecule adsorption energy in *K(T)*, whereas *RT* is used for molar thermodynamic quantities, with $R=N_{\textrm{A}}k_{\textrm{B}}$. As a practical descriptor of reversibility, we defined $T_{50}$ as the temperature at which half of the adsorbed $\hbox {H}_2$ is released under a reference pressure. A candidate was considered thermodynamically promising when $T_{50}$ fell within the U.S. Department of Energy (DOE) on-board hydrogen storage operating window of *-25* to $100\,^{\circ }$C^36^, indicating a balance between measurable room-temperature uptake and feasible desorption under near-ambient conditions. Within the present screening framework, the Langmuir/van’t Hoff treatment is used to compare relative thermodynamic trends across the dopant series and to identify candidates with the most balanced adsorption–desorption behaviour.

For decision-oriented visualization, we further classify systems using three heuristic screening thresholds: a broad target adsorption window of $|E_{\textrm{ads}}| = 0.2$–0.6 eV per $\hbox {H}_2$, a room-temperature coverage threshold of $\theta \ge 0.5$ at 298 K and 1 bar, and the DOE-aligned desorption window defined by $T_{50}=248$–373 K. These cutoffs are used for comparative screening and visualization only.

### Methodological scope and literature-grounded validation

The present calculations were designed as a comparative screening study on large, finite anatase $\hbox {TiO}_2$ nanoparticles rather than as an exhaustive higher-level adsorption benchmark on reduced slab or cluster models. In the present model, the pristine nanoparticle contains 269 atoms ($\hbox {Ti}_{{89}}$ $\hbox {O}_{{180}}$) and has a diameter of approximately 1.8 nm, while the adsorbate-bound system contains 271 atoms after inclusion of one $\hbox {H}_2$ molecule. This size was deliberately chosen to balance computational tractability with a realistic surface-to-bulk ratio; notably, approximately 63% of Ti atoms reside at surface positions (corner, edge, or terrace sites), making the particle representative of experimentally relevant ultrasmall anatase nanoparticles rather than an idealized bulk-like fragment. However, this physically meaningful system size also places exhaustive higher-level DFT optimization beyond the practical scope of a broad dopant-screening campaign. Repeating full geometry optimizations for chemically distinct pristine and $\hbox {H}_2$-adsorbed nanoparticles across the complete dopant series would be computationally prohibitive and would necessarily force a severe reduction in chemical coverage. We therefore employed DFTB+ with the PTBP Slater–Koster parameterization together with D3(BJ) dispersion corrections as a computationally tractable and internally consistent framework. This choice is consistent with the intended role of PTBP, which was introduced as a broadly transferable solid-state DFTB baseline with wide periodic-table coverage, including oxides, while not being positioned as a strict replacement for higher-level first-principles theory^32^.

To further validate the qualitative electronic trends obtained within the present screening framework, we compared the DFTB electronic-structure description against established literature for doped $\hbox {TiO}_2$ nanoparticles and against representative hybrid-DFT studies on doped anatase $\hbox {TiO}_2$^38,39^. Importantly, this comparison was made using the $\hbox {H}_2$-free nanoparticle gaps, since these are the quantities most directly comparable to reported optical gaps and bulk/slab electronic-structure data. Before considering dopant-specific effects, we note that the calculated pristine nanoparticle gap in the present work (3.5828 eV) remains within the expected wide-gap regime of nanoscale anatase $\hbox {TiO}_2$. This value should be interpreted as a finite-particle HOMO–LUMO/frontier-level gap obtained with the PTBP DFTB Hamiltonian, rather than as a quantity directly interchangeable with either a periodic bulk Kohn–Sham band gap or an experimental optical gap. This distinction is important because experimental optical gaps depend on optical-transition and excitonic effects, whereas the present frontier-level gap reflects the combined influence of finite-size confinement, surface termination, structural relaxation, and the underlying DFTB Hamiltonian.

This interpretation is further supported by the validation scope of the PTBP parameter set used here. PTBP was developed as a solid-state DFTB baseline by fitting to PBE reference energy–volume data and was benchmarked for transferability to multicomponent solid-state systems, including binary hydrides, nitrides, oxides, and fluorides^32^. Importantly for the present oxide nanoparticle series, $\hbox {TiO}_2$ is explicitly included in the binary oxide benchmark set of the PTBP study^32^. This validation strategy is also consistent with broader first-principles screening practice, where hydrogen adsorption free energies are commonly used as quantitative descriptors for comparing hydrogen reactivity trends across catalyst surfaces^40^. The magnitude of the pristine gap is also physically reasonable. Anatase $\hbox {TiO}_2$ is commonly associated with a bulk optical gap near 3.2 eV, while nanoparticle and nanostructured anatase samples can exhibit larger apparent gaps because of finite-size, surface, crystallinity, and measurement effects^41–43^. Reported anatase $\hbox {TiO}_2$ nanoparticle gaps include values in the 3.26–3.29 eV range for 25–50 nm particles, while smaller anatase nanoparticles and structurally constrained anatase systems show blue-shifted or enlarged apparent gaps^41–43^. Therefore, the 3.5828 eV value obtained here for a 1.8 nm finite nanoparticle is consistent with the expected wide-gap and size-affected character of nanoscale anatase $\hbox {TiO}_2$. The present dataset does not permit a unique numerical decomposition of this value into separate confinement and Hamiltonian contributions; such a decomposition would require a controlled size series or matched higher-level calculations on the same finite nanoparticles. We therefore use the gap as a screening-level electronic descriptor and as an internal reference for dopant-induced frontier-level changes.

Turning to dopant-dependent trends, weakly perturbing dopants such as Zr, Sn, and Nb generally preserve the host anatase character or induce only moderate band-edge shifts: Zr-doped $\hbox {TiO}_2$ nanoparticles have been reported to improve thermo-stability while retaining the anatase nanostructure^28^; Sn-doped $\hbox {TiO}_2$ remains phase-pure anatase while exhibiting broadened visible absorption and improved photoelectrochemical response rather than severe host-gap collapse^29^; and Nb-doped $\hbox {TiO}_2$ is commonly discussed in terms of a positive conduction-band shift and improved electron transport^30^. By contrast, more electronically disruptive dopants consistently show stronger gap narrowing and dopant-state formation: Fe-doped $\hbox {TiO}_2$ nanoparticles exhibit a red-shifted absorption edge with the optical gap reduced from 3.0 to 2.1 eV^44^; V-doped $\hbox {TiO}_2$ nanoparticles show a composition-dependent decrease from 3.18 to 2.60 eV^45^; Mo-doped $\hbox {TiO}_2$ nanoparticles have been reported to narrow from 3.48 to 2.79 eV, with theory attributing the effect to Mo 4d impurity states below the conduction band^46^, while complementary spectroscopy identifies charge transfer from Ti 3d to Mo 4d states together with local structural distortion in Mo-doped nanoparticles^47^; and W-doped $\hbox {TiO}_2$ nanoparticles are likewise reported to lower the band gap and alter phase behavior at higher loading^48^. Al-containing $\hbox {TiO}_2$ nanoparticle systems also show defect-mediated band-gap narrowing and lattice-distortion effects, although the resulting behavior is more appropriately described as moderate band-gap tuning than as the strongly perturbed Fe/V/Mo/W-type regime^49^. These external results do not constitute a quantitative benchmark of adsorption energetics, and we do not present them as such. Rather, they show that the qualitative electronic partitioning recovered by the present DFTB calculations, namely, a separation between weakly perturbing, host-preserving dopants and more strongly gap-narrowing dopants, is chemically consistent with both nanoparticle experiments and higher-level electronic-structure studies on doped anatase $\hbox {TiO}_2$^38,39^. Open-shell effects are expected to be most relevant for partially filled 3d dopants such as Fe and V. Spin-polarized DFT and DFT+U studies of transition-metal-doped anatase $\hbox {TiO}_2$ have shown that such dopants can introduce magnetic moments, spin-asymmetric density of states, and dopant-derived gap states^50,51^. We therefore treat Fe- and V-containing systems primarily as qualitative indicators of strongly perturbing, activation-prone chemistry rather than as spin-state-resolved quantitative benchmarks. This does not change the main screening outcome, because Fe is not selected as a balanced reversible-storage candidate, V remains loading-limited, and the most balanced candidates identified here, Nb and Zr, are not open-shell 3d dopants. Spin-polarized DFT or spin-resolved DFTB calculations on representative Fe- and V-doped structures would be the appropriate next step for refining their absolute adsorption energetics. Under this interpretation, the present DFTB workflow is used as a realistic screening tool for relative dopant ranking at system sizes where exhaustive higher-level optimization is presently impractical, not as a claim of one-to-one reproduction of absolute literature band gaps or adsorption energies. Possible systematic shifts in the absolute adsorption energies therefore cannot be excluded, but the qualitative conclusions are based on relative trends that are supported by the PTBP solid-state benchmark, the physically reasonable pristine $\hbox {TiO}_2$ nanoparticle gap, and the literature-consistent separation between weakly perturbing and strongly gap-narrowing dopants.

### Interpretable machine learning and active learning

To relate $\hbox {H}_2$ adsorption energetics to the electronic descriptor space, we employed two complementary machine-learning approaches: SR and GP regression. SR was used to identify compact analytical relationships between the CDFT descriptors and $E_{\textrm{ads}}$, thereby enabling physically interpretable descriptor–property analysis. In parallel, GP regression was used as a probabilistic surrogate model to predict adsorption energies and quantify the associated uncertainty in the small-data regime.

All descriptor inputs were standardized prior to GP fitting, and the resulting surrogate was coupled to an active-learning strategy in which new candidates were selected according to maximum posterior uncertainty. This workflow was used both to examine the data efficiency of the surrogate and to prospectively rank previously unseen dopant candidates for subsequent DFTB follow-up calculations.

Further details of the symbolic-regression setup, Gaussian-process regression, and active-learning protocol are provided in Section S1 of the Supplementary Information. Statistical reliability at *n = 11* was assessed using leave-one-out cross-validation (LOOCV) and a permutation null test with *K = 500* label shuffles to obtain an empirical RMSE distribution under the null hypothesis of no descriptor–property dependence. Symbolic-regression robustness was further evaluated via PySR^52^ complexity sweeps on the full dataset and LOOCV folds, reporting training and out-of-fold RMSE as a function of model complexity (Section S1).

## Results and discussion

**Figure 1:**
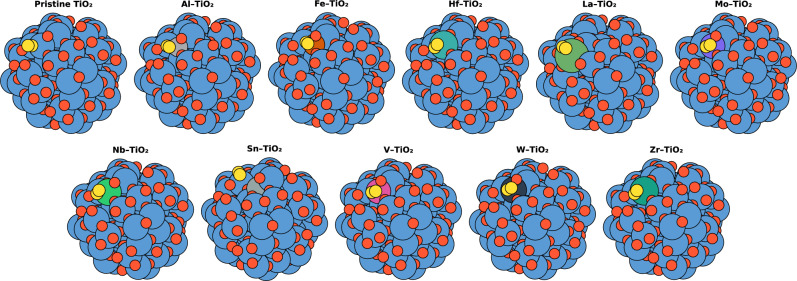
Optimized geometries of pristine TiO_2_-$\hbox {H}_2$ and doped TiO_2_-$\hbox {H}_2$ nanoparticles. Ti atoms are shown in blue, O in red, and H in yellow. Dopant atoms are individually colour-coded: Al (silver), Fe (orange), Hf (teal), La (green), Mo (violet), Nb (green), Sn (grey), V (pink), W (teal), and Zr (teal).

Hydrogen storage on oxide nanomaterials is governed not only by the strength of $\hbox {H}_2$ binding, but also by whether adsorption remains thermodynamically reversible within practical operating windows. Here, we integrate DFTB-based adsorption calculations, thermodynamic screening, and electronic descriptor analysis to compare $\hbox {H}_2$ adsorption on pristine and single-atom-doped anatase $\hbox {TiO}_2$ nanoparticles. By analyzing adsorption energies together with adsorption geometries, desorption midpoint temperatures ($T_{50}$), and descriptor-level changes in electronic reactivity, we distinguish the extent to which adsorption trends arise from local surface-site chemistry or from broader dopant-induced modifications of the electronic structure of the nanoparticle. These results provide the basis for identifying dopant chemistries that best balance uptake and deliverability for reversible hydrogen storage.

### Optimized structures and local binding geometries

Figure 1 summarizes the optimized geometries of the pristine and single-atom-doped anatase $\hbox {TiO}_2$ nanoparticles together with their corresponding $\hbox {H}_2$ adsorption configurations. The pristine $\hbox {TiO}_2$ nanoparticle adopts a compact, near-spherical morphology with a range of exposed Ti and O surface sites available for adsorption. Substitutional surface doping preserves the overall nanoparticle framework in all cases, while locally modifying the coordination environment around the adsorption region. This indicates that the dopants primarily perturb the local surface chemistry rather than inducing large-scale structural reconstruction of the nanoparticle.

The optimized structural metrics reveal that most systems retain predominantly molecular $\hbox {H}_2$ adsorption. In the pristine nanoparticle, the H–H bond length is 0.758 Å, and most doped systems remain close to this value, spanning from 0.766 Å for La to 0.814 Å for Mo. This behaviour indicates that, for the majority of dopants, adsorption occurs without substantial $\hbox {H}_2$ activation. The clearest exception is Fe-doped $\hbox {TiO}_2$, where the H–H bond is elongated to 0.981 Å, corresponding to an increase of nearly 0.22 Å relative to the pristine case and signalling incipient dissociative adsorption at the Fe site. Mo- and W-doped nanoparticles also exhibit moderate H–H elongation (0.814 and 0.791 Å, respectively), suggesting partial activation of the adsorbed molecule, although still within a largely molecular adsorption regime.

A similarly clear dopant dependence is observed in the adsorbate–site distance, $d_{\textrm{H}}$. The shortest separation is found for Fe-doped $\hbox {TiO}_2$ (1.460 Å), consistent with strong local interaction and the onset of chemisorptive activation, whereas La-doped $\hbox {TiO}_2$ shows the largest separation (2.631 Å), indicative of much weaker binding. Al-, V-, and W-doped nanoparticles display intermediate adsorbate–site distances in the range of approximately 1.9–2.0 Å, while Hf, Zr, Nb, and Sn cluster closer to 2.1–2.3 Å, remaining nearer to the pristine value of 2.009 Å. An instructive exception is the Sn-substituted nanoparticle, for which the preferred adsorption site is a neighbouring Ti atom rather than the dopant itself. This suggests that Sn modifies the surrounding surface environment without acting as the primary binding centre for $\hbox {H}_2$.

Taken together, the structural results define three adsorption regimes. La represents a weakly interacting limit with minimal perturbation of the $\hbox {H}_2$ molecule. Pristine $\hbox {TiO}_2$ together with Al, Hf, Nb, Sn, V, W, and Zr supports predominantly molecular adsorption with moderate site affinity. Fe occupies the opposite extreme, showing pronounced $\hbox {H}_2$ activation and clear incipient dissociation, while Mo lies in an intermediate regime between molecular adsorption and activated binding. These structural trends provide the first indication that dopant chemistry controls not only adsorption strength, but also the extent to which the surface promotes $\hbox {H}_2$ activation. This distinction is important because molecular $\hbox {H}_2$ adsorption and dissociative $\hbox {H}_2$ activation are different elementary processes. First-principles studies on anatase $\hbox {TiO}_2$-supported single-atom systems have shown that molecular adsorption, heterolytic splitting, homolytic splitting, and diffusion must be treated as separate pathway-dependent processes with local-environment-dependent energetics^53^. In the present work, the thermodynamic screening was constructed for molecular $\hbox {H}_2$ adsorption and reversible uptake–release behavior. The elongated H–H bond in Fe-doped $\hbox {TiO}_2$ therefore identifies Fe as an activation-prone outlier rather than as a balanced reversible molecular-storage candidate. No dissociation barriers were computed in the present screening study; resolving such pathways would require dedicated reaction-path calculations, such as nudged elastic band or constrained reaction-coordinate analyses. If dissociation were kinetically accessible for a given dopant, the Langmuir/van’t Hoff treatment would need to be extended to include molecular and dissociated surface states, dissociation/recombination barriers, and hydrogen chemical potentials. This does not alter the main molecular-adsorption screening conclusion, because the most balanced candidates identified here, Nb and Zr, retain molecular $\hbox {H}_2$ adsorption with H–H distances close to the pristine reference (see Table 1).

**Table 1:** $\hbox {H}_2$ adsorption structural metrics for the doped $\hbox {TiO}_2$ nanoparticles. $d_{\mathrm {H-H}}$: intramolecular H–H bond length (Å). $d_{\textrm{mid}}$: distance from the $\hbox {H}_2$ midpoint to the nearest surface atom. $d_{\textrm{H}}$: distance from the nearest H atom to the adsorption site. Site: element at the adsorption site.

System	$d_{\mathrm {H-H}}$ (Å)	$d_{\textrm{mid}}$ (Å)	$d_{\textrm{H}}$ (Å)	Site
Pristine	0.758	2.388	2.009	Ti
Al	0.775	1.940	1.974	Al
Fe	0.981	1.377	1.460	Fe
Hf	0.776	2.208	2.238	Hf
La	0.766	2.605	2.631	La
Mo	0.814	1.856	1.884	Mo
Nb	0.777	2.106	2.141	Nb
Sn	0.769	2.215	2.241	Ti
V	0.784	1.933	1.938	V
W	0.791	1.976	1.978	W
Zr	0.774	2.254	2.285	Zr

### Adsorption energetics

**Figure 2:**
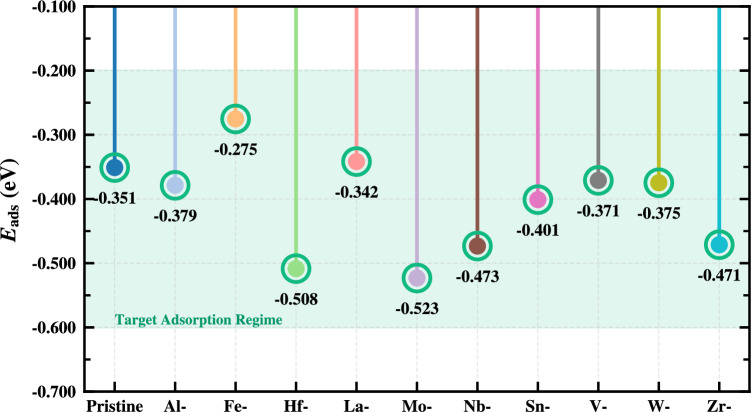
$\hbox {H}_2$ adsorption energies on pristine and doped $\hbox {TiO}_2$ nanoparticles. The green-shaded band denotes a heuristic adsorption-energy screening window often used to identify candidates for reversible $\hbox {H}_2$ adsorption (approximately *-0.2* to *-0.6* eV per $\hbox {H}_2$). This band is not a hard DOE specification; it is used here only as a visual guide, while reversibility is assessed using the Langmuir/van’t Hoff thermodynamic analysis and the corresponding $T_{50}$ values.

Figure 2 shows that single-atom doping produces a substantial variation in $\hbox {H}_2$ adsorption strength across the anatase $\hbox {TiO}_2$ nanoparticle series, with $E_{\textrm{ads}}$ spanning more than 0.25 eV, from *-0.275* eV for Fe to *-0.523* eV for Mo. This wide distribution demonstrates that dopant chemistry provides an effective handle for tuning the balance between $\hbox {H}_2$ uptake and reversibility on the $\hbox {TiO}_2$ surface.

Notably, all eleven systems fall within a broad heuristic adsorption-energy window often used for preliminary screening of reversible $\hbox {H}_2$ adsorption ($-0.60 \le E_{\textrm{ads}} \le -0.20$ eV per $\hbox {H}_2$). This interval is not a DOE specification and should not be interpreted as a universal optimum; the preferred binding strength depends on the assumed pressure–temperature swing and adsorption mechanism. For example, Bhatia and Myers derived an optimum hydrogen adsorption enthalpy of about 15.1 kJ mol$^{-1}$ for ambient-temperature delivery between 30 and 1.5 bar, while broader computational studies emphasize that stronger metal–$\hbox {H}_2$ interactions can remain relevant for reversible near-ambient adsorption depending on the operating window^54,55^. Even within this broad regime, however, the dopants span a wide range of binding strengths that translates into markedly different practical performance, as shown by the thermodynamic screening below. Mo (*-0.523* eV) and Hf (*-0.509* eV) define the strong-binding edge, suggesting the highest room-temperature uptake but more demanding release conditions. Nb (*-0.473* eV) and Zr (*-0.471* eV) occupy an intermediate range and offer the most balanced compromise between adsorption strength and practical reversibility. Sn (*-0.401* eV), Al (*-0.379* eV), W (*-0.375* eV), and V (*-0.371* eV) cluster near the middle of the window, where moderate binding favours easier desorption but may limit uptake.

At the weak-binding end, pristine $\hbox {TiO}_2$ (*-0.351* eV) and La (*-0.342* eV) interact only weakly with $\hbox {H}_2$, consistent with their relatively long adsorbate–site distances and limited local electronic perturbation. Fe-doped $\hbox {TiO}_2$ exhibits the weakest adsorption energy in the series (*-0.275* eV), despite showing the strongest structural signature of $\hbox {H}_2$ activation. This indicates that local bond activation at the Fe site does not translate into a thermodynamically stable molecular adsorption state, but instead reflects an incipient transition toward dissociative interaction. Thus, although all systems satisfy the broad adsorption-energy criterion, the thermodynamic analysis below shows that only a subset combines useful uptake with release under practical operating conditions.

Overall, these results show that single-atom doping can shift the $\hbox {H}_2$ adsorption energy of anatase $\hbox {TiO}_2$ nanoparticles over a chemically meaningful range and place multiple candidates in a practically relevant regime for reversible hydrogen storage. In particular, Nb and Zr emerge as the most balanced dopants in the present set, whereas Mo and Hf represent stronger-binding alternatives for applications where enhanced uptake is prioritised. Stoichiometric $\hbox {TiO}_2$ single-crystal surfaces often exhibit very weak molecular $\hbox {H}_2$ adsorption, so the broader adsorption range found here is consistent with the greater structural and chemical diversity of nanoparticle surface sites relative to ideal flat terraces^6,10,11^.

**Table 2:** CDFT descriptors and $\hbox {H}_2$-adsorption descriptor shifts for $\hbox {TiO}_2$ nanoparticles doped with the listed element (Al-, Fe-, ...) and the undoped (Pristine) reference, all with $\hbox {H}_2$ adsorbed. Paired columns show the bare-nanoparticle value with the percentage change upon $\hbox {H}_2$ adsorption in parentheses. The percentage shift is defined as $\Delta X(\%) = 100 \times [X_{\mathrm {H_2 ads}}-X_{\textrm{bare}}]/|X_{\textrm{bare}}|$. With this convention, for negative frontier orbital energies, a negative *Δ* indicates a shift to more negative energy upon $\hbox {H}_2$ adsorption, whereas a positive *Δ* indicates an upward shift toward less negative energy.

System	($E_\textrm{H}$ (*Δ*)	$E_\textrm{L}$ (*Δ*)	$E_g$ (*Δ*)	IP	EA	*μ*	*η* (*Δ*)	*S* (*Δ*)	*ω* (*Δ*)	*χ* (*Δ*)	$\omega ^-$	$\omega ^+$	$\Delta N_{\max }$
Pristine	$-8.116\;(-0.00\%)$	$-4.533\;(-0.00\%)$	$3.583\;(+0.00\%)$	8.116	4.533	*-6.325*	$1.791\;(+0.00\%)$	$0.279\;(+0.00\%)$	$11.165\;(+0.00\%)$	$6.325\;(+0.00\%)$	18.385	5.736	1.765
Al-	$-7.351\;(-0.03\%)$	$-4.501\;(-0.00\%)$	$2.850\;(-0.07\%)$	7.351	4.501	*-5.926*	$1.425\;(-0.07\%)$	$0.351\;(+0.07\%)$	$12.323\;(+0.04\%)$	$5.926\;(-0.02\%)$	18.962	7.109	2.079
Fe-	$-7.001\;(-6.91\%)$	$-6.439\;(-8.27\%)$	$0.562\;(+8.68\%)$	7.001	6.439	*-6.720*	$0.281\;(+8.68\%)$	$1.779\;(-7.99\%)$	$80.315\;(-21.37\%)$	$6.720\;(-7.56\%)$	87.175	73.736	11.952
Hf-	$-8.118\;(-0.01\%)$	$-4.532\;(-0.08\%)$	$3.586\;(+0.08\%)$	8.118	4.532	*-6.325*	$1.793\;(+0.08\%)$	$0.279\;(-0.08\%)$	$11.156\;(-0.14\%)$	$6.325\;(-0.03\%)$	18.378	5.728	1.764
La-	$-8.115\;(-0.08\%)$	$-4.534\;(-0.06\%)$	$3.581\;(-0.10\%)$	8.115	4.534	*-6.324*	$1.791\;(-0.10\%)$	$0.279\;(+0.10\%)$	$11.168\;(-0.04\%)$	$6.324\;(-0.07\%)$	18.387	5.739	1.766
Mo-	$-5.973\;(-1.46\%)$	$-5.097\;(-2.73\%)$	$0.876\;(+5.92\%)$	5.973	5.097	*-5.535*	$0.438\;(+5.92\%)$	$1.141\;(-5.59\%)$	$34.964\;(-9.41\%)$	$5.535\;(-2.04\%)$	40.718	29.649	6.317
Nb-	$-8.116\;(-0.00\%)$	$-4.321\;(-0.10\%)$	$3.795\;(+0.11\%)$	8.116	4.321	*-6.219*	$1.897\;(+0.11\%)$	$0.264\;(-0.11\%)$	$10.191\;(-0.18\%)$	$6.219\;(-0.03\%)$	17.359	4.921	1.639
Sn-	$-8.117\;(-0.00\%)$	$-4.534\;(-0.00\%)$	$3.583\;(-0.00\%)$	8.117	4.534	*-6.325*	$1.792\;(-0.00\%)$	$0.279\;(+0.00\%)$	$11.166\;(+0.00\%)$	$6.325\;(-0.00\%)$	18.387	5.737	1.765
V-	$-5.384\;(-2.57\%)$	$-4.821\;(-3.51\%)$	$0.563\;(+5.40\%)$	5.384	4.821	*-5.103*	$0.282\;(+5.40\%)$	$1.775\;(-5.12\%)$	$46.221\;(-10.75\%)$	$5.103\;(-3.01\%)$	51.464	41.259	9.058
W-	$-5.614\;(-1.97\%)$	$-4.533\;(-0.00\%)$	$1.081\;(-10.23\%)$	5.614	4.533	*-5.074*	$0.540\;(-10.23\%)$	$0.925\;(+11.40\%)$	$23.815\;(+8.98\%)$	$5.074\;(-1.09\%)$	29.159	19.012	4.694
Zr-	$-8.118\;(-0.01\%)$	$-4.532\;(-0.06\%)$	$3.586\;(+0.06\%)$	8.118	4.532	*-6.325*	$1.793\;(+0.06\%)$	$0.279\;(-0.06\%)$	$11.154\;(-0.12\%)$	$6.325\;(-0.03\%)$	18.376	5.726	1.764

**Figure 3:**
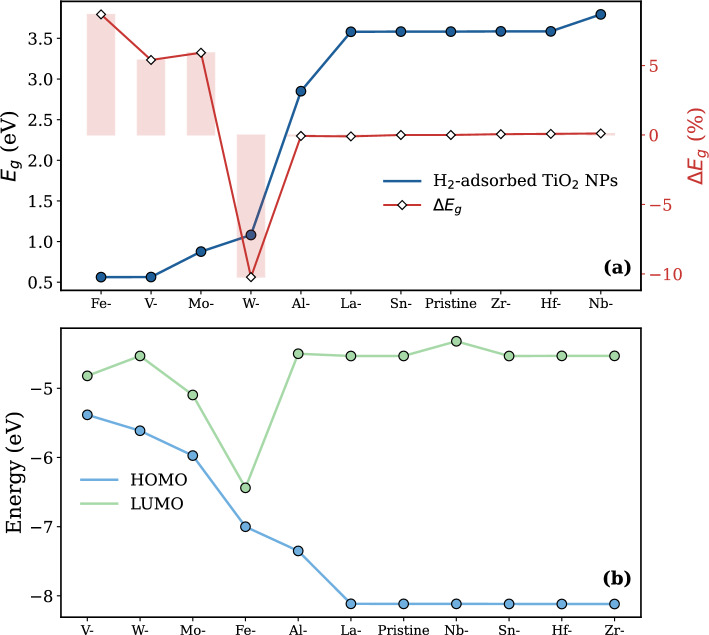
Frontier-orbital characteristics of the $\hbox {H}_2$-adsorbed $\hbox {TiO}_2$ nanoparticles. (**a**) $E_g$ values arranged from low to high together with the corresponding percentage change, $\Delta E_g$. (**b**) $E_\textrm{H}$ and $E_\textrm{L}$ energies arranged independently from high to low according to the $E_\textrm{H}$ energy.

#### Decision-oriented classification of $\hbox {H}_2$ storage operability

**Figure 4:**
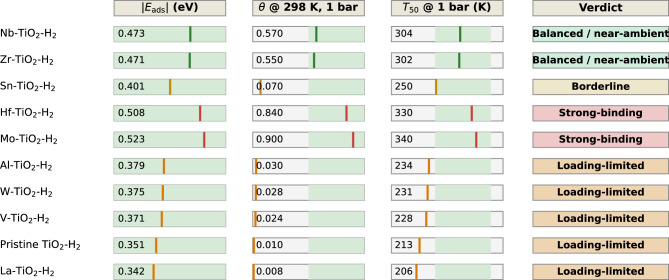
Decision-oriented screening panel for $\hbox {H}_2$ storage on pristine and doped $\hbox {TiO}_2$ nanoparticles. Systems are compared using adsorption strength, room-temperature coverage, and desorption midpoint temperature at 1 bar. The shaded target regions correspond to the broad adsorption window ($|E_{\textrm{ads}}| = 0.2$–0.6 eV), higher room-temperature coverage ($\theta \ge 0.5$), and the DOE operating window for $T_{50}$ (248–373 K).

To summarize the practical implications of the adsorption and thermodynamic results, the pristine and doped $\hbox {TiO}_2$ nanoparticles were further organized into a decision-oriented storage panel based on adsorption strength, room-temperature coverage, and desorption midpoint temperature at 1 bar (Fig. 4). This representation provides a compact operability-oriented view of the dataset and helps distinguish dopants that remain within a balanced near-ambient storage window from those that move toward strong-binding or loading-limited regimes. Within this classification, Nb- and Zr-doped $\hbox {TiO}_2$ emerge as the most balanced candidates, combining favorable adsorption energies with appreciable room-temperature coverage and desorption temperatures close to ambient conditions. Sn occupies a borderline position, since its $T_{50}$ value remains within the targeted window while its room-temperature coverage is substantially lower. By contrast, Hf- and Mo-doped $\hbox {TiO}_2$ fall on the strong-binding side of the distribution, whereas Al-, W-, V-, La-doped, pristine, and Fe-doped $\hbox {TiO}_2$ are more appropriately classified as loading-limited under near-ambient conditions. The panel therefore complements the scalar descriptor analysis by translating the calculated energetics and thermodynamics into a more application-oriented screening summary.

#### Frontier orbital energies and HOMO–LUMO gap

Table 2 compiles the CDFT descriptors for the pristine and doped $\hbox {TiO}_2$ nanoparticles with $\hbox {H}_2$ adsorbed, while Figure 3 visualizes the corresponding frontier-orbital energies and HOMO–LUMO gaps. Because anatase $\hbox {TiO}_2$ is intrinsically a wide-gap oxide with frontier levels that are relatively well separated, changes in the $E_\textrm{H}$ and $E_\textrm{L}$ upon doping provide a direct measure of how strongly the dopant perturbs the low-energy electronic structure of the host lattice^6^. In the present dataset, a clear electronic partitioning emerges across the dopant series. Hf, La, Zr, Nb, and Sn preserve frontier orbital energies that remain essentially indistinguishable from those of the pristine nanoparticle, with $E_\textrm{H}$ values clustered near *-8.1* eV, $E_\textrm{L}$ values near *-4.5* eV, and wide $E_g$ of approximately 3.58 eV. Nb slightly increases the gap to 3.795 eV through an upward shift of the $E_\textrm{L}$ to *-4.321* eV, whereas Al produces a more visible narrowing to 2.850 eV by raising the $E_\textrm{H}$ to *-7.351* eV. Despite this reduction, Al still remains electronically distinct from the narrow-gap transition-metal-doped systems and does not exhibit the pronounced collapse of the pristine gap. The limited frontier-level perturbation observed for several dopants is also consistent with previous reports that not all substitutions in $\hbox {TiO}_2$ produce strong frontier-level reorganization; in many cases, the dominant effect remains local rather than global^56,57^.

This wide-gap subgroup is important because it indicates that not all substitutional dopants qualitatively reorganize the $\hbox {TiO}_2$ electronic framework. Instead, several dopants act mainly as local chemical modifiers while leaving the broader frontier-orbital structure of the nanoparticle largely intact. Such behaviour is consistent with previous electronic-structure studies showing that some non-transition-metal or rare-earth substitutions perturb the band edges only modestly, or contribute weakly to the frontier region compared with more strongly hybridizing transition-metal dopants^58,59^. In particular, La behaves here as a weakly perturbing dopant, in line with literature showing that La-derived states may contribute only weakly near the frontier region depending on the substitutional environment, even when rare-earth incorporation modifies the broader electronic landscape^59^.

In contrast, Fe, V, Mo, and W form a second class characterized by strong gap narrowing. Fe- and V-doped $\hbox {TiO}_2$ exhibit nearly identical HOMO–LUMO gaps of 0.562 and 0.563 eV, respectively, indicating an almost complete collapse of the wide-gap electronic structure found in pristine $\hbox {TiO}_2$. Mo and W also show strongly reduced gaps of 0.876 and 1.081 eV, respectively, placing them in the same narrow-gap regime even though their electronic perturbation is somewhat less extreme than for Fe and V. As shown in Figure 3(a), these four dopants are cleanly separated from the remaining systems when ranked by $E_g$, confirming that transition-metal substitution can dramatically reshape the low-energy electronic structure of the nanoparticle.

The physical origin of this behaviour is consistent with a large body of electronic-structure work on doped $\hbox {TiO}_2$. For 3*d* dopants such as V and Fe, ab initio calculations have shown that substitution can create localized occupied states inside the pristine band gap, with the dopant-derived levels shifting downward in energy across the transition series; in particular, V- and Fe-doped $\hbox {TiO}_2$ are known to form mid-gap states rather than simply preserving the pristine frontier-level^58^. Likewise, for group-6 dopants such as Mo and W, previous first-principles studies have reported the emergence of gap states and substantial band-gap reduction, with the relevant electronic structure arising from hybridized O-2*p*, Ti-3*d*, and dopant-*d* contributions^60^. The present DFTB results follow precisely this qualitative pattern: the strongest gap collapse is observed for those dopants most likely to inject dopant-derived states into the pristine $\hbox {TiO}_2$ gap.

The frontier-orbital trends in Figure 3(b) further clarify the origin of this partitioning. For the wide-gap systems, both $E_\textrm{H}$ and $E_\textrm{L}$ remain close to the pristine reference, indicating that the electronic response to adsorption is largely governed by the host $\hbox {TiO}_2$ framework. By contrast, Fe, V, Mo, and W shift one or both frontier levels deep into the pristine band gap, consistent with the formation of dopant-derived mid-gap states. These states are most naturally attributed to partially filled transition-metal *d* orbitals and provide the electronic basis for the enhanced softness, electrophilicity, and adsorption reactivity observed for the narrow-gap dopants. From a CDFT perspective, this marked reduction in $E_g$ is expected to correlate with lower hardness and higher softness, which is exactly the trend observed in the descriptor analysis reported below^26,27^. In this sense, the frontier-orbital analysis shows that dopant chemistry does not merely tune adsorption strength quantitatively, but can qualitatively reorganize the electronic structure of the $\hbox {H}_2$-adsorbed nanoparticle.

#### Total density of states of representative strongly perturbing dopants

**Figure 5:**
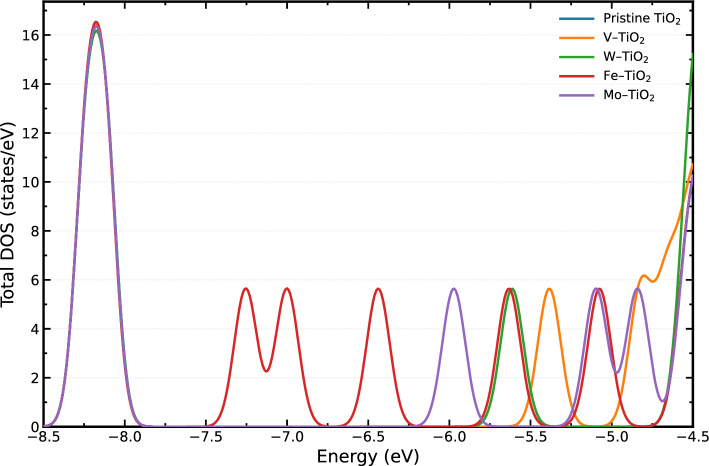
TDOS of the pristine $\hbox {TiO}_2$ nanoparticle and representative strongly perturbing doped systems (V, W, Fe, and Mo) in the selected energy window (*-8.5* to *-4.5* eV). The doped nanoparticles show clear redistribution of states relative to pristine $\hbox {TiO}_2$, consistent with the narrow-gap regime.

To complement the frontier-orbital analysis, the total density of states (TDOS) of the pristine $\hbox {TiO}_2$ nanoparticle was compared with representative doped systems that exhibited the largest gap reduction, namely V-, W-, Fe-, and Mo-doped $\hbox {TiO}_2$ (Fig. 5). Only these most strongly perturbed cases are shown for clarity, since the primary purpose of this comparison is to visualize the electronic restructuring associated with the narrow-gap regime identified from the descriptor analysis in Table 2.

As seen in Fig. 5, all systems retain the dominant deeper-lying valence feature centered near *-8.2* eV, indicating that the overall oxide valence framework is not removed by doping. However, relative to pristine $\hbox {TiO}_2$, the doped nanoparticles develop distinct additional DOS features in the higher-energy valence / frontier region between approximately *-7.5* and *-4.5* eV. These extra features are particularly pronounced for Fe- and Mo-doped $\hbox {TiO}_2$, while V- and W-doped systems also display clear redistribution of spectral weight within the same energy window. Thus, the reduced HOMO–LUMO gaps observed for these systems are not merely isolated numerical changes, but are accompanied by substantial dopant-dependent modification of the frontier electronic structure.

Among the selected cases, Fe-doped $\hbox {TiO}_2$ shows the most extensive restructuring over the widest portion of the plotted energy range, with multiple new peaks emerging well above the main pristine valence feature. Mo-doped $\hbox {TiO}_2$ likewise exhibits pronounced additional states at intermediate energies, while V- and W-doped $\hbox {TiO}_2$ show more localized but still clearly resolved DOS perturbations closer to the frontier region. This behavior is fully consistent with the literature-based classification discussed above, in which Fe, V, Mo, and W act as electronically more disruptive dopants than host-preserving cases such as Zr, Sn, or Nb. Importantly, the TDOS results indicate that the narrowed-gap regime arises from dopant-induced redistribution of states near the frontier levels rather than from a complete breakdown of the underlying $\hbox {TiO}_2$ valence structure.

Fig. 5 therefore provides qualitative support for the electronic-regime separation inferred from the descriptor analysis. The dopants identified as strongly perturbing in Table 2 also exhibit the most pronounced redistribution of electronic states in the frontier-energy region relative to pristine $\hbox {TiO}_2$, consistent with their narrowed-gap character.

#### Chemical hardness, softness, and electrophilicity

The chemical hardness *η* closely mirrors the frontier-orbital partitioning discussed above, which is expected because, within the CDFT framework, hardness is directly linked to the energetic separation between occupied and unoccupied frontier levels. In wide-gap oxide systems such as anatase $\hbox {TiO}_2$, large frontier-level separation generally corresponds to electronically rigid, weakly polarizable surfaces, whereas dopant-induced gap narrowing is typically accompanied by a marked softening of the electronic response^6,26,27^. This trend is clearly borne out in the present dataset. The wide-gap group (Pristine, Hf, La, Zr, Sn, and Nb) clusters at $\eta \approx 1.79$–1.90 eV, indicating chemically hard nanoparticles with limited charge deformability. Al is moderately softer (*η = 1.425* eV), consistent with its partially reduced $E_g$ but still distinct from the strongly perturbed transition-metal-doped systems. By contrast, the narrow-gap dopants are markedly softer: W (*η = 0.540* eV), Mo (*η = 0.438* eV), and Fe and V ($\eta \approx 0.28$ eV). The global softness *S* follows the expected inverse trend, reaching 1.779 $\hbox {eV}^{-1}$ for Fe and 1.775 $\hbox {eV}^{-1}$ for V, roughly six times the pristine value.

This separation between hard and soft electronic regimes is chemically significant. In the wide-gap subgroup, the $\hbox {TiO}_2$ host largely retains its intrinsic oxide-like character, and adsorption is therefore expected to be governed by relatively modest charge redistribution. In contrast, the softened response of Fe-, V-, Mo-, and W-doped nanoparticles points to a much more deformable frontier-electron manifold, which can respond more strongly to the presence of the adsorbed $\hbox {H}_2$ molecule. Such behaviour is consistent with prior electronic-structure studies showing that transition-metal doping in $\hbox {TiO}_2$ can introduce dopant-derived states inside the pristine gap, thereby lowering the effective electronic stiffness of the host. In particular, V- and Fe-doped $\hbox {TiO}_2$ have long been associated with localized mid-gap states derived from dopant *d* orbitals, while Mo- and W-substituted systems likewise show substantial gap reduction and strong hybridization near the frontier region^60,58^. The present descriptor trends therefore align well with the broader literature: the dopants that most strongly collapse $E_g$ are also those that produce the largest reduction in hardness and the strongest increase in softness.

This softness amplification, in turn, strongly enhances the electrophilicity index *ω*. Pristine $\hbox {TiO}_2$ and the wide-gap dopants exhibit $\omega \approx 10$–12 eV, consistent with a moderate electron-accepting tendency. The narrow-gap systems, however, show a dramatic escalation: W (*ω = 23.8* eV), Mo (*ω = 35.0* eV), V (*ω = 46.2* eV), and Fe (*ω = 80.3* eV). Because *ω* depends quadratically on the chemical potential and inversely on the hardness, this sharp increase is a natural consequence of the dopant-induced softening described above. In physical terms, the narrow-gap dopants generate adsorption sites that are much more responsive to incoming electron density and therefore much more capable of stabilizing charge rearrangement during $\hbox {H}_2$ interaction.

The donor–acceptor asymmetry indices $\omega ^-$ and $\omega ^+$ further refine this picture. Fe-doped $\hbox {TiO}_2$ exhibits by far the largest values for both electron acceptance ($\omega ^- = 87.2$ eV) and back-donation ($\omega ^+ = 73.7$ eV), identifying Fe as the electronically most reactive surface environment in the entire dataset. This result is fully consistent with the structural signatures discussed above, where Fe also shows the strongest H–H elongation and the clearest onset of dissociative activation. The maximum fractional electron transfer $\Delta N_{\max }$ follows the same hierarchy, peaking at 11.95 for Fe and 9.06 for V, compared with *∼*1.76 for the pristine nanoparticle. These elevated $\Delta N_{\max }$ values indicate a much stronger driving force for charge redistribution upon adsorbate contact and support the interpretation that narrow-gap dopants create far more electronically labile adsorption centres.

Taken together, the hardness, softness, and electrophilicity descriptors show that the dopant series separates into two chemically distinct regimes. Hf, La, Zr, Sn, Nb, and, to a lesser extent, Al retain the harder and less polarizable character of the $\hbox {TiO}_2$ host, whereas Fe, V, Mo, and W create electronically soft, strongly electrophilic surfaces. This distinction is central for understanding the adsorption behaviour observed here: dopants that produce the strongest softening also exhibit the most pronounced signatures of $\hbox {H}_2$ activation and the largest deviation from purely host-controlled adsorption chemistry. In this sense, the CDFT descriptors do not merely restate the frontier-orbital analysis, but translate it into a more direct chemical language of charge-transfer propensity and adsorption reactivity^26,27^.

#### Descriptor response to $\hbox {H}_2$ adsorption

The percentage shifts *Δ* induced by $\hbox {H}_2$ adsorption provide a useful complementary measure of adsorbate–surface coupling strength. Whereas the absolute descriptor values discussed above distinguish between wide-gap and narrow-gap electronic regimes, the adsorption-induced changes reveal how strongly the electronic structure responds once the molecule is actually bound. Within a CDFT framework, small changes in frontier levels, hardness, and electrophilicity imply that adsorption perturbs the host only weakly, as expected for predominantly molecular, physisorption-like binding. Larger descriptor shifts, in contrast, indicate stronger charge redistribution, greater adsorbate-induced polarization, and a more pronounced reorganization of the local frontier-electron manifold^26,27^.

This distinction is clearly visible in the present dataset. Pristine $\hbox {TiO}_2$ and Sn-$\hbox {TiO}_2$ show essentially negligible descriptor changes ($|\Delta | < 0.01\%$ across all quantities), fully consistent with their weak adsorption response and the absence of significant electronic restructuring upon $\hbox {H}_2$ binding. Hf, La, Zr, and Nb likewise display only marginal perturbations ($|\Delta | \le 0.1\%$), indicating that although these dopants tune $E_{\textrm{ads}}$ into a practically relevant regime, they do so without substantially reorganizing the electronic structure of the $\hbox {TiO}_2$ host. In other words, these systems improve adsorption primarily through modest local chemical tuning rather than through strong frontier-level reconstruction.

The narrow-gap dopants respond far more strongly. Fe-doped $\hbox {TiO}_2$ undergoes the largest changes in the entire series: $E_\textrm{H}$ and $E_\textrm{L}$ deepen by 6.91% and 8.27%, respectively, while $E_g$ widens by 8.68% and the electrophilicity *ω* decreases by 21.37%. This pronounced electronic reorganization is consistent with the strong local adsorbate–surface interaction inferred from the structural data, particularly the substantial H–H elongation and the onset of dissociative activation at the Fe site. V and Mo follow a qualitatively similar trend, showing gap widening (*+5.4%* and *+5.9%*), softness reduction (*-5.1%* and *-5.6%*), and electrophilicity decrease (*-10.8%* and *-9.4%*), albeit with smaller amplitudes than Fe. These changes indicate that adsorption partially quenches the extreme softness of the unbound narrow-gap state by stabilizing the adsorbate-bound electronic configuration, while still preserving a much stronger response than in the wide-gap subgroup.

This behaviour is physically reasonable in light of the electronic structure established above. Fe, V, and Mo already introduce dopant-derived states near or inside the pristine $\hbox {TiO}_2$ gap, and such states are expected to couple more strongly to an incoming adsorbate than the comparatively rigid frontier-level structure of the pristine host. As a result, adsorption on these systems does not simply add a weak molecular perturbation, but instead modifies the occupation and energetic position of the frontier region itself. This interpretation is consistent with previous theoretical work showing that doped or metal-modified $\hbox {TiO}_2$ surfaces can exhibit much stronger $\hbox {H}_2$ interaction and more pronounced adsorbate-driven electronic rearrangement than the pristine oxide, especially when dopant-derived states participate directly in the binding process^60,61,58^.

W-doped $\hbox {TiO}_2$ is a notable outlier. It is the only system in which $E_g$ narrows upon adsorption (*-10.23%*), accompanied by an increase in softness (*+11.40%*) and electrophilicity (*+8.98%*). Rather than partially quenching the narrow-gap state as in Fe, V, and Mo, $\hbox {H}_2$ adsorption on W appears to further stabilize or reinforce the dopant-derived frontier-state manifold. This qualitatively distinct response suggests a different coupling mechanism in which adsorption enhances, rather than attenuates, the electronic softness of the active site. Although the structural activation of $\hbox {H}_2$ on W is less extreme than on Fe, the descriptor response indicates that the W site remains highly electronically labile and may follow a different adsorption pathway from the rest of the narrow-gap subgroup.

Taken together, the adsorption-induced descriptor shifts confirm that the dopant series separates not only into different static electronic classes, but also into different dynamical response classes upon $\hbox {H}_2$ binding. Pristine $\hbox {TiO}_2$ together with Sn, Hf, La, Zr, and Nb behaves as a weakly perturbed host, where adsorption leaves the electronic structure largely intact. Fe, V, Mo, and W, by contrast, exhibit strong adsorption-induced electronic reorganization, reflecting much stronger coupling between the adsorbate and dopant-derived frontier states. In this sense, the percentage descriptor response serves as a direct probe of how actively each dopant site participates in the electronic stabilization of adsorbed $\hbox {H}_2$, complementing the trends derived from adsorption energies, geometries, and the static CDFT descriptor values.

### Thermodynamic screening

**Figure 6:**
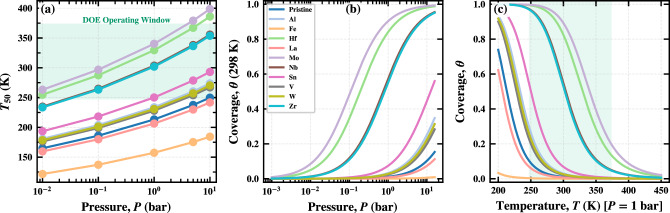
Thermodynamic performance screening for pristine and doped $\hbox {TiO}_2$ nanoparticles. (**a**) Pressure-dependent desorption midpoint temperature, $T_{50}$; the green-shaded band denotes the DOE target operating window (248–373 K). (**b**) Predicted $\hbox {H}_2$ surface coverage, *θ*, as a function of pressure at 298 K based on the Langmuir isotherm model. (**c**) Predicted *θ* as a function of temperature at 1 bar; the green-shaded band again marks the DOE operating window.

*Desorption midpoint temperature.*

Figure 6(a) translates the adsorption-energy trends into the desorption midpoint temperature $T_{50}$ as a function of pressure. For all systems, $T_{50}$ increases with pressure, as expected from the equilibrium shift toward the adsorbed state at higher $\hbox {H}_2$ fugacity. The dopant series spans a broad thermodynamic range: at 1 bar, Fe-doped $\hbox {TiO}_2$ gives the lowest $T_{50}$ value of 157 K, whereas Mo-doped $\hbox {TiO}_2$ reaches the highest at 340 K. Five systems fall within the DOE operating window at 1 bar, namely Mo (340 K), Hf (330 K), Nb (304 K), Zr (302 K), and Sn (250 K).

Within this subset, Mo and Hf define the strong-binding side of the thermodynamic window. Both systems enter the DOE range only at sub-atmospheric pressures and exceed its upper bound above roughly 5 bar, indicating that practical operation would require careful pressure control. By contrast, Nb and Zr remain comfortably within the target range across the full 0.1–10 bar interval, making them the most robustly operable candidates in the present screening set. Sn (250 K) sits only slightly above the lower DOE threshold, indicating more marginal compliance and stronger sensitivity to pressure. Al ($T_{50}=234$ K) lies just below the lower bound, whereas the pristine nanoparticle ($T_{50}=213$ K), together with La (206 K), V (228 K), and W (231 K), falls more clearly below the target window, showing that these systems release $\hbox {H}_2$ too readily for practical ambient-condition storage. Fe lies furthest below the desired range ($T_{50}=157$ K), which is fully consistent with its anomalously weak molecular adsorption state and its tendency toward incipient dissociative interaction.

*Coverage–pressure behaviour at room temperature.*

Figure 6(b) shows the predicted $\hbox {H}_2$ surface coverage *θ* as a function of pressure at 298 K based on the Langmuir isotherm model. In all cases, coverage increases monotonically with pressure, but the magnitude of uptake varies dramatically across the dopant series. Mo and Hf display the strongest room-temperature uptake, reaching *θ = 0.90* and 0.84 at 1 bar, respectively, and approaching saturation (*θ > 0.98*) by 10 bar. Nb and Zr follow closely, with $\theta \approx 0.57$ and 0.55 at 1 bar and values above 0.93 at 10 bar. These four dopants therefore combine thermodynamically accessible desorption temperatures with substantial room-temperature loading, which is a prerequisite for useful hydrogen storage.

Sn (*θ = 0.07* at 1 bar) and Al (*θ = 0.03*) occupy a more marginal position. Their comparatively weaker adsorption leads to much lower room-temperature uptake, and meaningful coverage develops only at elevated pressure. The remaining systems show negligible storage performance at 298 K: pristine $\hbox {TiO}_2$ gives *θ = 0.01*, La 0.008, V 0.024, W 0.028, and Fe only 0.0006. For these weakly binding surfaces, thermal energy at room temperature is sufficient to overcome the adsorption well, making them ineffective for practical ambient-condition storage.

*Coverage–temperature behaviour at 1 bar.*

Figure 6(c) reports the corresponding temperature dependence of *θ* at 1 bar, providing a direct view of the usable loading–release swing across the DOE operating window. At the lower bound of the window (248 K), Mo, Hf, Nb, and Zr all retain nearly full coverage (*θ > 0.96*), whereas at the upper bound (373 K) their coverage decreases to 0.20, 0.14, 0.05, and 0.05, respectively. The resulting coverage swing, $\Delta \theta _{248\text {--}373}$, quantifies the fraction of stored hydrogen that can be released within the target operating interval. Mo gives $\Delta \theta \approx 0.80$, Hf 0.85, and both Nb and Zr about 0.92. Although Nb and Zr exhibit the largest swing, this should be interpreted together with their already moderate room-temperature uptake, so that deliverable capacity is governed by both the initial loading and the release fraction.

Sn and Al also display fractional swings (0.53 and 0.29, respectively), but they begin from much lower absolute coverage, which limits their practical deliverable capacity. By contrast, the weakly binding systems (pristine, La, Fe, V, and W) are already substantially depleted at 248 K (*θ < 0.3*), leaving little hydrogen available for controlled release within the DOE window. Taken together, Figure 6(b,c) provides a coherent thermodynamic picture: among the eleven systems screened, Mo, Hf, Nb, and Zr emerge as the most promising candidates, combining appreciable room-temperature uptake with effective release under DOE-relevant conditions. Sn remains thermodynamically viable but a lower-capacity alternative, whereas Al and the remaining dopants fall outside the practical storage regime at 1 bar. From a hydrogen-storage perspective, these results also highlight the importance of balancing adsorption strength against deliverability, rather than maximizing binding alone^54^.

### Machine-learning-guided screening

The machine-learning analysis was used to assess descriptor–property relationships, surrogate-model data efficiency, and prospective candidate ranking across the dopant series.

**Figure 7:**
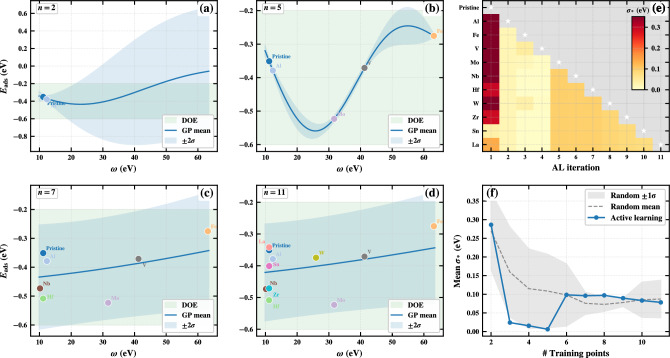
Active-learning convergence of the GP surrogate model. A greedy maximum-uncertainty acquisition strategy iteratively selects $\hbox {TiO}_2$ dopant candidates for DFTB evaluation. The GP surrogate is trained on all CDFT descriptors; panels (**a**)–(**d**) show one-dimensional posterior projections onto the electrophilicity index *ω* at *n=2*, 5, 7, and 11 training points, with the GP mean (blue line), the $\pm 2\sigma$ uncertainty band (shaded), and the DOE-relevant adsorption-energy window (green band). Panel (**e**) shows the full uncertainty heatmap $\sigma (\textrm{step},\textrm{element})$ across all active-learning iterations; NaN entries denote candidates already incorporated, and asterisks mark the greedy selection at each step. Panel (f) compares the mean posterior uncertainty of the active-learning strategy (solid line) against 50 random-selection trials (mean ± std, shaded), demonstrating that uncertainty-guided sampling reduces surrogate uncertainty more rapidly than random baselines during the early stages, with both strategies converging as the training set becomes exhaustive.

#### Active Learning Convergence

*Surrogate model construction*

A GP surrogate was trained on the CDFT descriptor space using a composite kernel (constant *×* RBF + white noise) together with a greedy maximum-uncertainty acquisition strategy. The pristine $\hbox {TiO}_2$ nanoparticle was used as the initial seed, and the first acquisition added Al; accordingly, the *n=2* projection in Figure 7(a) corresponds to the {Pristine, Al} training set. At this stage, the posterior is tightly constrained only in the low-*ω* region near the initial points and remains highly uncertain in the high-electrophilicity regime populated by Fe, V, and Mo. The acquisition strategy therefore selects Fe, then V, and then Mo, rapidly covering the electronically distinct narrow-gap region of descriptor space. By *n=5*, the GP mean already captures the non-monotonic relationship between *ω* and $E_{\textrm{ads}}$, and the $\pm 2\sigma$ band contracts substantially. Subsequent acquisitions refine the densely populated low-*ω* cluster, where dopants with similar electrophilicity still exhibit meaningfully different adsorption energies. By *n=11*, the posterior uncertainty collapses onto the training data, indicating that the surrogate has fully resolved the descriptor space represented in the current benchmark set.

*Convergence and sampling efficiency*

The uncertainty heatmap in Figure 7(e) provides a system-resolved view of surrogate improvement across the active-learning sequence. At the beginning of the procedure, unsampled candidates carry comparably large posterior uncertainty. Once Fe is incorporated, the model sharply reduces uncertainty across much of the low-*ω* cluster, while still identifying V and W as comparatively uncertain and therefore informative next acquisitions. After only five training points, the mean posterior uncertainty drops dramatically, indicating that the surrogate has already learned the dominant structure of the descriptor–property landscape. A transient increase in uncertainty at intermediate steps reflects the redistribution of posterior variance as the model incorporates electronically distinct outliers and then rebalances uncertainty within the remaining low-*ω* region.

Figure 7(f) benchmarks the greedy strategy against 50 random-selection trials. During the early stages (*n łe 5*), uncertainty-guided sampling consistently achieves lower mean posterior uncertainty than random acquisition, demonstrating that the model identifies the most informative candidates substantially more efficiently than an unguided baseline. Beyond this point, both strategies converge as the training set approaches exhaustion. This early-stage advantage is practically important: the active-learning workflow reaches surrogate performance sufficient for DOE-window classification after only five DFTB evaluations, i.e., with fewer than half of the full dataset.

Leave-one-out cross-validation at *n = 11* (Fig. S2) yields LOOCV RMSEs of 0.087 eV for the twelve CDFT descriptors and 0.101 eV for the five elemental features (electronegativity, ionic radius, *d*-electron count, oxidation state, atomic mass) on a 0.248 eV target spread, with $R^{2} = -0.39$ and *-0.79* respectively. A permutation null test (*K = 500* label shuffles) returns one-sided *p*-values of 0.751 and 0.790, so the GP mean is not statistically distinguishable from a constant predictor at this sample size. We therefore use the GP exclusively as a sampling-design tool: the posterior uncertainty field $\sigma (\textbf{x})$ depends only on training-point geometry and remains a legitimate basis for active-learning acquisition, but the predicted means $\mu (\textbf{x})$ should not be read as quantitative estimates of $E_{\textrm{ads}}$.

#### Symbolic regression and descriptor importance

*Pareto-front models*

SR was used to identify closed-form expressions linking the CDFT descriptor space to the $\hbox {H}_2$ adsorption energy, $E_{\textrm{ads}}$. The expressions below are compact structural decompositions of the descriptor–property data rather than causal models. Figure S1(a,b) compares parity plots for two representative models selected from the Pareto front. At low complexity (*C = 5*), SR recovers the simple expression $E_{\textrm{ads}} = -0.467 + 0.00212\,\omega ^{-}$ ($R^2 = 0.21$), identifying the electron-donating power $\omega ^{-}$ as the most informative single descriptor but fails to capture the non-monotonic structure of the full dataset. In particular, the model substantially underestimates Mo ($E_{\textrm{ads}}^{\textrm{DFTB}} = -0.523$ eV, $E_{\textrm{ads}}^{\textrm{SR}} = -0.387$ eV) and Hf (*-0.509* vs *-0.428* eV), while overestimating the pristine nanoparticle (*-0.351* vs *-0.428* eV). The largest residuals occur within the low-$\omega ^{-}$ cluster, where a spread of *∼ 0.17* eV in adsorption energy among dopants with nearly identical $\omega ^{-}$ values cannot be resolved by a one-descriptor model.

**Figure 8:**
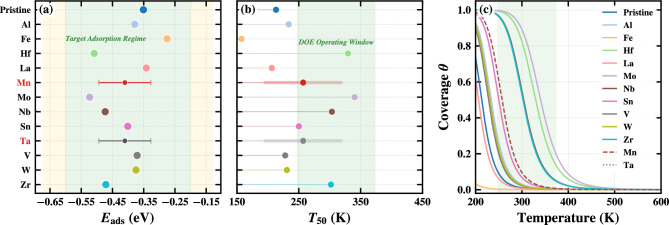
GP-based screening and thermodynamic viability of candidate dopants for $\hbox {H}_2$ storage on $\hbox {TiO}_2$ nanoparticles. Panel (**a**) shows GP-predicted adsorption energies, $E_\textrm{ads}$, for the candidate set (Mn, Ta) together with the DFTB-computed training values (filled circles); error bars indicate the GP posterior uncertainty ($\pm \sigma$). The green band marks the DOE-relevant adsorption-energy window, and the yellow band denotes a marginal zone around the target desorption range. Panel (**b**) ranks all systems by their half-coverage desorption temperature, $T_{50}$, at *P=1* bar; training systems are shown as solid markers and candidates as open markers, with $T_{50}$ uncertainty intervals propagated from the GP $\pm \sigma$ bounds on $E_\textrm{ads}$. Panel (c) displays Langmuir coverage isotherms, *θ (T)*, at *P=1* bar; training systems are shown as solid curves and candidates (Mn, Ta) as dashed curves. Mn and Ta share identical predicted $E_{\textrm{ads}} \approx -0.41$ eV and posterior uncertainty $\sigma \approx 0.08$ eV; the greedy acquisition tie-breaker selects Mn as the top next-acquisition target.

At higher complexity (*C = 28*, the highest non-dominated point on the PySR Pareto front at maximum complexity 30), the expression introduces a nonlinear dependence on *μ*, HOMO, $\Delta N_{\max }$, EA, and $E_{\textrm{L}}$, raising the in-sample fit to $R^2 = 0.70$ and reproducing the narrow-gap systems (Fe, Mo, V) to within *∼ 11* meV, with the largest residuals on Hf (91 meV) and Zr (54 meV). These are in-sample residuals on the full *n = 11* set; a leave-one-out complexity sweep (Fig. S3) shows that training RMSE decreases smoothly from 0.074 to 0.044 eV across the Pareto front, whereas LOOCV RMSE stays at 0.08–0.14 eV for *C łe 16* before jumping to 0.38–1.6 eV beyond *C = 17*. The *C = 28* expression therefore lies deep in the overfitting regime and is presented in Fig. S1 as an in-sample structural decomposition only, while the *C = 5* $\omega ^{-}$-only expression is retained as the LOOCV-stable interpretable model. This is consistent with the descriptor-correlation analysis (Section Conceptual DFT Descriptors, Fig. S4), where two principal components already capture more than *95%* of the descriptor variance, so the effective dimensionality is far below the nominal twelve.

*Feature sensitivity analysis*

Figure S1(c,d) sweeps each descriptor across its training range with the others held at their means. At *C = 5* only $\omega ^{-}$ contributes ($|\Delta E_{\textrm{ads}}| = 0.11$ eV); at *C = 28*, $\Delta N_{\max }$ dominates, followed by HOMO and *μ*, with smaller contributions from $E_{\textrm{L}}$ and EA. The magnitude of the $\Delta N_{\max }$ sensitivity reflects the chosen symbolic form rather than a measurable adsorption-energy variation, so only the relative descriptor ranking is interpreted. The active descriptors at *C = 28* span chemical-potential, frontier-orbital, and charge-transfer information, consistent with the dopants that most strongly perturb $E_{\textrm{ads}}$ (Fe, Mo, V, W) being those that shift *μ* and HOMO; given the overfitting at *C = 28* established above, this is read as a structural decomposition rather than predictive accuracy on unseen dopants.

#### Candidate screening and thermodynamic viability

*GP-predicted adsorption energies*

The trained GP surrogate was used to predict $E_{\textrm{ads}}$ for two untested candidates, Mn and Ta, chosen as the unseen-dopant elements whose CDFT-descriptor vectors lie closest to the training manifold under the SR Pareto-front model (Section 3.4.1). As shown in Figure 8(a), both fall within the DOE-relevant adsorption window, with essentially identical predicted means $E_{\textrm{ads}} \approx -0.410$ eV and posterior uncertainties $\sigma \approx 0.083$ eV. The tie reflects the limited discriminative power of the elemental-feature surrogate at *n = 10* (see Section 3.4.2 and Fig. S2); the greedy acquisition rule selects Mn as the top next-acquisition target, with Ta an equally informative alternative.

*Desorption temperature ranking*

Figure 8(b) places these candidate predictions in the context of the full thermodynamic ranking at 1 bar. Within the training set, Mo, Hf, Nb, Zr, and Sn fall inside the target DOE window (248–373 K), whereas Al, W, V, Pristine, La, and Fe lie below the desired range. Both candidates enter this ranking with mean $T_{50}$ inside the target window and identical uncertainty intervals ($\sigma _{T_{50}} \approx 60$ K) propagated from the GP $\pm \sigma$ on $E_{\textrm{ads}}$.

*Coverage isotherms*

Figure 8(c) shows the corresponding Langmuir coverage isotherms, *θ (T)*, at 1 bar. The training systems reproduce the same three-tier thermodynamic behaviour identified above: Hf and Mo retain high coverage to relatively high temperatures, the balanced systems (notably Nb, Zr, Sn, and Al) show substantial loading together with effective release across the DOE window, and the weakest-binding systems are depleted at lower temperature. The candidate isotherms (dashed curves) cluster closely together and converge near $\theta \approx 0.103$ at 298 K, indicating similar usable loading under ambient conditions. Their placement near the behaviour of the viable training systems suggests that the GP surrogate is successfully identifying thermodynamically relevant candidates across chemically distinct regions of descriptor space.

*Reading the small-data ML results* At *n = 11* the GP mean is not statistically distinguishable from a constant baseline (Section 3.4.1, Fig. S2), so the active-learning ranking should be understood as an uncertainty-driven exploration heuristic that prioritizes candidates lying farthest from the training manifold, not as a quantitative prediction of $E_{\textrm{ads}}$. The SR expressions are LOOCV-stable only up to *C ≈ 16* (Fig. S3); beyond this they overfit and should not be treated as predictive. Within these limits, the descriptor analysis robustly identifies the design-relevant axes – electrophilicity, frontier-orbital energies, and gap-related quantities – and the GP provides a defensible procedure for prioritizing the next dopant chemistries for higher-level computation.

## Conclusions

In this work, we developed a descriptor-guided workflow for screening $\hbox {H}_2$ adsorption on pristine and single-atom-doped anatase $\hbox {TiO}_2$ nanoparticles by integrating DFTB calculations, conceptual-DFT descriptors, thermodynamic analysis, symbolic regression, Gaussian-process modelling, and active learning. Across the dopant series, single-atom substitution tuned the adsorption energy over a chemically meaningful range, from *-0.275* eV for Fe to *-0.523* eV for Mo, indicating that dopant identity can modulate both the strength and character of $\hbox {H}_2$ binding on $\hbox {TiO}_2$ nanoparticles. Structural and electronic analyses further distinguished weakly perturbed systems, which largely retain molecular adsorption and the wide-gap character of the $\hbox {TiO}_2$ host, from more strongly perturbed cases such as Fe, V, Mo, and W, where dopant-derived frontier states are associated with enhanced softness and electrophilicity. Within the machine-learning analysis, electrophilicity emerged as the most informative descriptor for adsorption energetics, with the HOMO–LUMO gap, electron affinity, and LUMO energy providing additional nonlinear contributions, while active learning showed that the main descriptor–property relationships can be recovered with relatively few additional DFTB calculations. These ML conclusions should be read at the resolution the *n = 11* benchmark supports. The leave-one-out complexity sweep shows that symbolic expressions remain LOOCV-stable up to *C ≈ 16*, so the robust SR output is the identification of electrophilicity and frontier-orbital descriptors as the dominant design axes rather than the precise numerical sensitivities of higher-complexity expressions. The Gaussian-process surrogate is correspondingly best used as an uncertainty-driven sampling-design tool for active learning rather than a point-prediction model, an interpretation supported by a permutation null test in which the GP point error is not statistically distinguishable from label-shuffled chance (*p ≈ 0.75* for both feature sets; Section 3.4.1). Thermodynamic screening also showed that adsorption energy alone is not sufficient to identify practically relevant hydrogen-storage candidates, since viability depends on achieving a balanced uptake–release profile within the DOE-relevant operating window. Under this criterion, Nb and Zr provide the most balanced behaviour in the present benchmark set, Sn remains a borderline but viable case, and Hf and Mo represent stronger-binding alternatives with less favourable release characteristics.

## Supplementary Information

Supplementary Information.

## Data Availability

The datasets generated and analyzed during this study are available in the Hydrogent GitHub repository: https://github.com/KurbanIntelligenceLab/Hydrogent.

## Code availability

The code used to generate the thermodynamic and machine-learning analyses in this study is available in the Hydrogent GitHub repository under the MIT License: https://github.com/KurbanIntelligenceLab/Hydrogent.
