# Supplementary material for: Descriptor-guided thermodynamic screening of [image] adsorption on single-atom-doped anatase [image] nanoparticles with interpretable machine learning
Source: Sci Rep. 2026 May 27;16:24203. doi: 10.1038/s41598-026-55149-w (PMC13442862; doi:10.1038/s41598-026-55149-w)
Supplement: Supplementary file 1 [file 41598_2026_55149_MOESM1_ESM.pdf]

Supplementary Information

for

Descriptor-guided thermodynamic screening of H_2 adsorption on single-atom-doped anatase TiO_2 nanoparticles with interpretable machine learning

Mustafa Kurban[a,b], Can Polat[c], Erchin Serpedin[c], Hasan Kurban[d]

[a] *Department of Prosthetics and Orthotics, Ankara University, Ankara, Turkey*
[b] *Department of Electrical and Computer Engineering, Texas A&M University at Qatar, Doha, Qatar*
[c] *Department of Electrical and Computer Engineering, Texas A&M University, College Station, TX, USA*
[d] *College of Science and Engineering, Hamad Bin Khalifa University, Doha, Qatar*

S1. Details of the Interpretable Machine-Learning Workflow

The machine-learning analysis presented in the main manuscript was designed to connect dopant-dependent H_2 adsorption energetics to a compact, physically interpretable electronic-descriptor space under limited-data conditions. All descriptors used in this workflow were derived from DFTB calculations performed with DFTB+ and the PTBP Slater–Koster parameter set, consistent with the computational setup described in the main text. The descriptor space itself was based on conceptual DFT (CDFT) quantities, which provide a chemically interpretable representation of global reactivity in terms of frontier-orbital energetics, charge-transfer propensity, and electrophilic or nucleophilic character. To relate these descriptors to adsorption energies, we employed two complementary machine-learning approaches: symbolic regression (SR) for interpretable descriptor–property discovery and Gaussian process (GP) regression for probabilistic prediction and uncertainty quantification. The target variable throughout was the H_2 adsorption energy, E_{ads}, and the input space consisted of the twelve CDFT descriptors defined in the main text.

S1.1. Symbolic Regression

SR was used to search for compact analytical expressions linking the descriptor vector $\mathbf{x}$ to the target property $\mathrm{y} = E_{\mathrm{ads}}$. For this purpose, we employed PySR (Cranmer, 2023), which uses a multi-population evolutionary search strategy and returns a Pareto set of candidate expressions ranked by predictive accuracy and symbolic complexity.

Given a feature matrix $\mathbf{X} \in \mathbb{R}^{n\times d}$ and target vector $\mathbf{y} \in \mathbb{R}^n$, the SR problem can be written as the minimization of the empirical risk over the space of symbolic expressions $\mathcal{F}$:

$$f^* = \underset{f\in\mathcal{F}}{\arg\min}\, \frac{1}{n}\sum_{i=1}^{n} \left(y_i - f(\mathbf{x}_i)\right)^2 . \tag{1}$$

To preserve interpretability, PySR does not return a single model by default but instead constructs a Pareto front of non-dominated solutions. A symbolic model f belongs to the Pareto set $\mathcal{P}$ if there exists no alternative expression g with both lower complexity and lower loss:

$$\begin{aligned}\mathcal{P} = \big\{ f \in \mathcal{F} \;\big|\; \nexists\, g \in \mathcal{F} : \;& C(g) \le C(f) \,\wedge \\ & \mathcal{L}(g) \le \mathcal{L}(f), \\ & \text{with at least one strict inequality} \big\},\end{aligned} \tag{2}$$

where $C(f)$ denotes the symbolic complexity (measured as expression tree size) and $\mathcal{L}(f)$ denotes the mean squared error. The final expression discussed in the main text was selected from this Pareto front as the simplest model that retained satisfactory predictive performance.

In the present implementation, the allowed binary operators were $\{+, -, \times, /\}$ and the unary operators were {square, neg}, where $\mathrm{square}(x) = x^2$ and $\mathrm{neg}(x) = -x$. The maximum symbolic complexity was set to 30 nodes, and the search was run for 500 iterations with a population count of 15. Mean squared

error was used as the optimization loss throughout.

S1.2. Gaussian Process Regression

GP regression was used as a probabilistic surrogate model to predict E_{ads} and to quantify the associated uncertainty for each candidate in descriptor space. In contrast to deterministic regression, the GP places a prior distribution over functions and updates it using the observed data to produce a posterior predictive mean and variance (Rasmussen and Williams, 2006).

We used a GP with a radial basis function (RBF) kernel augmented by a white-noise term:

$$k(\mathbf{x}_i, \mathbf{x}_j) = \sigma_f^2 \exp\left(-\frac{\|\mathbf{x}_i - \mathbf{x}_j\|^2}{2\ell^2}\right) + \sigma_n^2 \delta_{ij}, \tag{3}$$

where σ_f^2 is the signal variance, ℓ is the characteristic length scale, and σ_n^2 is the noise variance.

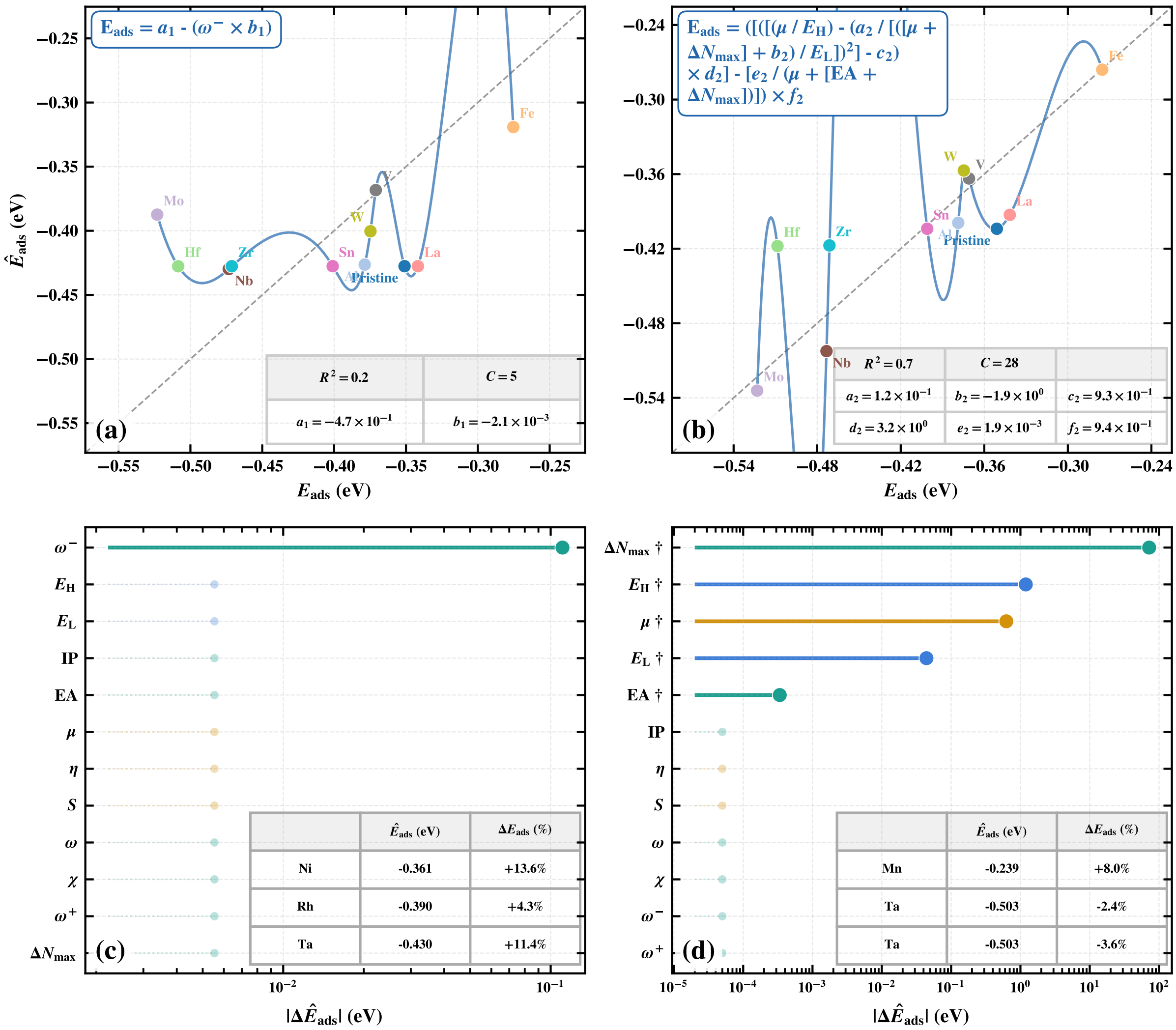

Figure S1: Symbolic-regression (SR) analysis of H_2 adsorption on doped TiO_2 nanoparticles. Panels (a) and (b) compare DFTB-computed and SR-predicted adsorption energies for representative low- and higher-complexity models ($C = 5$, $R^2 = 0.21$; $C = 28$, $R^2 = 0.70$, where $C = 28$ is the highest non-dominated point on the Pareto front returned by PySR at maximum complexity 30), respectively; dashed lines indicate perfect agreement. Panels (c) and (d) show the corresponding feature sensitivities, showing that ω^- dominates at low complexity, whereas additional descriptors contribute non-trivially in the higher-complexity model. The $C = 28$ panels report *in-sample* fit on the full $n = 11$ training set; a leave-one-out complexity sweep (Section S1.5 and Fig. S3) shows that this expression falls deep inside the overfitting regime, and the apparent high sensitivity to $\Delta N_{\max}$ in panel (d) reflects the chosen symbolic form rather than a directly measurable adsorption-energy variation. Symbolic regression establishes statistical correlation, not causation.

For a training set $(\mathbf{X}, \mathbf{y})$ and a test point $\mathbf{x}_*$, the posterior predictive mean and variance are given by

$$\mu_*(\mathbf{x}_*) = \mathbf{k}_*^\top \left(\mathbf{K} + \sigma_n^2 \mathbf{I}\right)^{-1} \mathbf{y}, \tag{4}$$

$$\sigma_*^2(\mathbf{x}_*) = k(\mathbf{x}_*, \mathbf{x}_*) - \mathbf{k}_*^\top \left(\mathbf{K} + \sigma_n^2 \mathbf{I}\right)^{-1} \mathbf{k}_*, \tag{5}$$

where $\mathbf{K}$ is the covariance matrix over the training points and $\mathbf{k}_*$ is the covariance vector between the test point and the training set.

The kernel hyperparameters $\boldsymbol{\theta} = \{\sigma_f^2, \ell, \sigma_n^2\}$ were determined by maximizing the log marginal likelihood,

$$\begin{aligned} \log p(\mathbf{y} \mid \mathbf{X}, \boldsymbol{\theta}) = & -\tfrac{1}{2}\mathbf{y}^\top \left(\mathbf{K} + \sigma_n^2 \mathbf{I}\right)^{-1} \mathbf{y} \\ & - \tfrac{1}{2}\log\left|\mathbf{K} + \sigma_n^2 \mathbf{I}\right| - \tfrac{n}{2}\log 2\pi, \end{aligned} \tag{6}$$

which balances data fit and model smoothness in a Bayesian manner. Because the descriptor set contains quantities with different scales and units, all input features were standardized to zero mean and unit variance prior to GP fitting using `StandardScaler` from scikit-learn (Pedregosa et al., 2011).

The trained GP was used to predict adsorption energies and posterior uncertainties for previously unseen dopant candidates, which were then ranked for prospective follow-up screening.

S1.3. Active Learning Protocol

To emulate sequential screening under limited computational budget, the GP surrogate was coupled to an active-learning protocol (Settles, 2009; Lookman et al., 2019). At each iteration, the next candidate was selected using the maximum-uncertainty (max-σ) acquisition rule,

$$\mathbf{x}_{\text{next}} = \arg\max_{\mathbf{x} \in \mathcal{C}} \sigma_*(\mathbf{x}), \tag{7}$$

where $\mathcal{C}$ denotes the current candidate pool and $\sigma_*(\mathbf{x})$ is the GP posterior standard deviation. This criterion prioritizes exploration of the least certain regions of the descriptor space and is therefore appropriate for the present small-data regime.

The information gained by selecting a candidate $\mathbf{x}_*$ can be viewed in terms of the reduction in posterior variance across the full candidate set:

$$\Delta\mathcal{I}(\mathbf{x}_*) = \sum_{\mathbf{x} \in \mathcal{C}} \left[\sigma_*^2(\mathbf{x}) - \sigma_{*|\mathbf{x}_*}^2(\mathbf{x})\right], \tag{8}$$

where $\sigma_{*|\mathbf{x}_*}^2(\mathbf{x})$ denotes the posterior variance after hypothetically incorporating the observation at $\mathbf{x}_*$. Although this information gain is not evaluated explicitly during acquisition, the max-σ rule provides a simple greedy approximation that preferentially selects candidates expected to reduce global uncertainty most strongly.

The active-learning loop proceeded as follows: (i) train the GP on the current dataset, (ii) predict E_{ads} and $\sigma_*(\mathbf{x})$ for all remaining candidates, (iii) select the candidate with the highest posterior uncertainty, (iv) evaluate that candidate by DFTB, and (v) augment the training set and retrain the GP. This procedure was used to assess how rapidly surrogate quality improves as new adsorption calculations are added sequentially.

S1.4. Gaussian-process validation under $n = 11$

To quantify the statistical reliability of the Gaussian-process surrogate at the present sample size, we performed leave-one-out cross-validation (LOOCV) on the two feature sets used in the main text: the twelve CDFT descriptors employed for descriptor–property modelling, and the five elemental features (electronegativity, ionic radius, *d*-electron count, oxidation state, atomic mass) used to project onto unseen dopant chemistries in active learning. On each fold the GP was retrained, including kernel-hyperparameter optimization, on the remaining $n - 1$ samples and used to predict the held-out point.

Table S1 summarizes the LOOCV metrics. The CDFT surrogate yields a LOOCV RMSE of 0.087 eV against a target spread of 0.248 eV, with $R^2 = -0.39$ and mean posterior standard deviation $\langle\sigma\rangle = 0.080$ eV; the five-elemental-feature surrogate yields RMSE $= 0.101$ eV, $R^2 = -0.79$, $\langle\sigma\rangle = 0.084$ eV. Both negative R^2 values indicate that, at $n = 11$, neither surrogate outperforms a constant-mean predictor on held-out systems.

To rule out the possibility that the LOOCV RMSE reflects merely the data spread rather than

Table S1: Leave-one-out cross-validation metrics for the Gaussian-process surrogate on the two feature sets used in this work.

Feature set	n	RMSE (eV)	R^2	$\langle\sigma\rangle$ (eV)
Twelve CDFT descriptors	11	0.087	−0.39	0.080
Five elemental properties	10	0.101	−0.79	0.084

any descriptor–property signal, we performed a permutation null test in which the target labels were randomly shuffled $K = 500$ times. For each shuffle, the LOOCV protocol was repeated end-to-end to generate an empirical distribution of LOOCV RMSEs under the null hypothesis of no descriptor–property relationship. The one-sided p-value, defined as the fraction of null replicates whose RMSE is at least as low as the observed RMSE, was $p = 0.751$ for the CDFT surrogate and $p = 0.790$ for the elemental surrogate. The observed point-prediction errors are therefore not statistically distinguishable from those obtained under randomly permuted labels at this sample size.

Figure S2 visualizes both validation analyses: panels (a) and (b) show LOOCV parity for the CDFT and elemental surrogates respectively, and panels (c) and (d) show the corresponding permutation null distributions with the observed RMSE marked. We emphasize that this is not a deficiency of the GP per se but a property of the descriptor–property problem at $n = 11$; the implication is that the GP should be read as a sampling-design tool whose posterior uncertainty $\sigma(\mathbf{x})$ encodes training-point geometry in feature space, rather than as a quantitative point-prediction model.

S1.5. Symbolic-regression complexity sweep

To assess the overfitting risk of the symbolic-regression analysis, we performed a leave-one-out complexity sweep: PySR was fit on the full dataset to provide the headline Pareto-front equations, and was refit on each of the eleven LOOCV folds. For each Pareto-front complexity C, we evaluated the fold's best equation with complexity $\leq C$ on the held-out row and aggregated the per-fold predictions into a single LOOCV RMSE at that complexity.

Figure S3 reports both the training RMSE (computed from the full-data Pareto front evaluated at each complexity) and the LOOCV RMSE as a function of C. The training RMSE decreases monotonically and smoothly from 0.074 eV at $C = 1$ to 0.044 eV at $C \geq 22$, the expected behaviour of a flexible regressor whose in-sample fit improves with capacity. The LOOCV RMSE, however, exhibits a pronounced cliff: it stays predominantly in the range 0.08–0.14 eV for $C \leq 16$, with isolated excursions reaching $\approx$ 0.25 eV at $C = 10$, and then jumps abruptly to $\approx$ 0.38–1.6 eV at $C \geq 17$, with the failure becoming progressively more catastrophic at higher complexity. The lowest LOOCV RMSE (corresponding to the most generalizing model) occurs at $C = 1$, with the lowest non-trivial generalizing complexity being $C \approx 5$.

S1.6. Descriptor correlation structure and effective dimensionality

Although the methods text and machine-learning pipeline employ twelve distinct CDFT descriptors, the descriptors are not statistically independent: most pairs of conceptual-DFT global reactivity indices are algebraic functions of the same underlying ionization potential, electron affinity, and frontier orbital energies. To quantify the resulting redundancy, we computed the pairwise Pearson correlation matrix of the twelve descriptors over the doped systems used in the ML analyses (Fig. S4a). 42 off-diagonal pairs exhibit $|r| > 0.7$, reflecting the algebraic relationships among χ, μ, η, S, ω, ω^-, ω^+, and $\Delta N_{\max}$ (all derivable from IP and EA), as well as the strong empirical coupling between E_{H} and E_{L}.

A principal-component analysis on the standardized descriptor matrix (Fig. S4b) shows that two components account for more than 95% of the descriptor variance and three components account for more than 99%. The effective dimensionality of the descriptor space relevant to this dataset is therefore approximately two to three, far smaller than the nominal count of twelve. This redundancy is the reason that the symbolic-regression Pareto front contains compact one-descriptor expressions at low complexity (Fig. S1a), and it materially relaxes the data-to-feature ratio that would otherwise apply to a sample 11.

References

Aradi, B.; Hourahine, B.; Frauenheim, T. DFTB+, a Sparse Matrix-Based Implementation of the DFTB Method. *J. Phys. Chem. A* **2007**, *111*, 5678–5684.

Cranmer, M. Interpretable Machine Learning for Science with PySR and SymbolicRegression.jl. *arXiv* **2023**, arXiv:2305.01582.

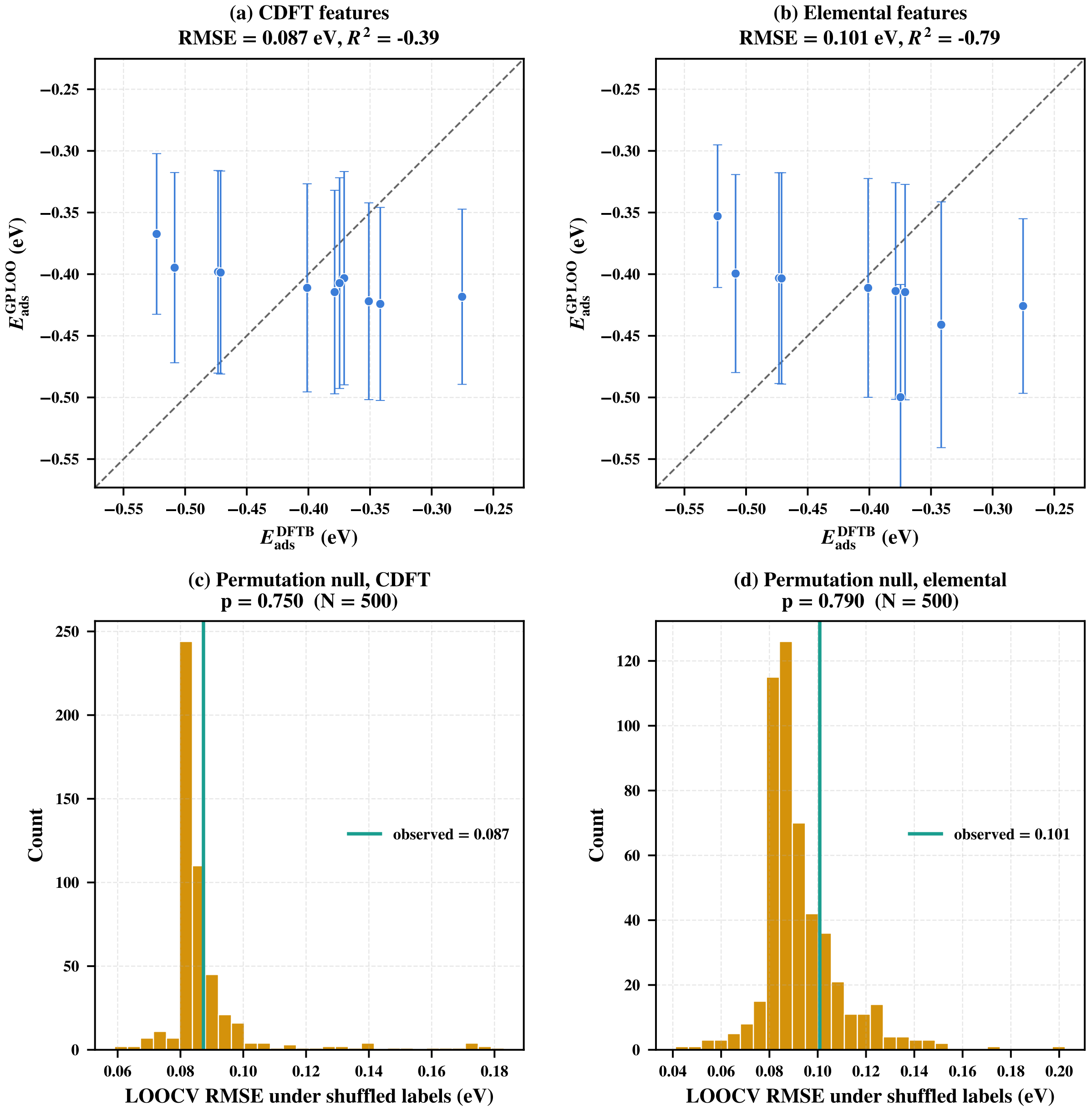

Figure S2: Validation of the Gaussian-process surrogate under $n = 11$. Panels (a) and (b): LOOCV parity plots for the twelve-CDFT-descriptor surrogate and the five-elemental-feature surrogate, with vertical bars showing the predictive σ at each held-out point. Panels (c) and (d): permutation null distributions (histograms of LOOCV RMSE under $K = 500$ label shuffles) with the observed RMSE marked by the vertical line and the one-sided p-value annotated. The observed RMSE lies near the centre of the null distribution for both feature sets, indicating that the GP point error is not distinguishable from label-shuffled chance at this sample size.

Cui, M.; Reuter, K.; Margraf, J. T. Obtaining Robust Density Functional Tight-Binding Parameters for Solids across the Periodic Table. *J. Chem. Theory Comput.* **2024**, *20*, 5276–5290.

Geerlings, P.; De Proft, F.; Langenaeker, W. Conceptual Density Functional Theory. *Chem. Rev.* **2003**, *103*, 1793–1874.

Lookman, T.; Balachandran, P. V.; Xue, D.; Yuan, R. Active Learning in Materials Science with Emphasis on Adaptive Sampling Using Uncertainties for Targeted Design. *npj Comput. Mater.* **2019**, *5*, 21.

Parr, R. G.; Szentpály, L. V.; Liu, S. Electrophilicity Index. *J. Am. Chem. Soc.* **1999**, *121*, 1922–1924.

Pedregosa, F.; Varoquaux, G.; Gramfort, A.; Michel, V.; Thirion, B.; Grisel, O.; Blondel, M.; Pretten-

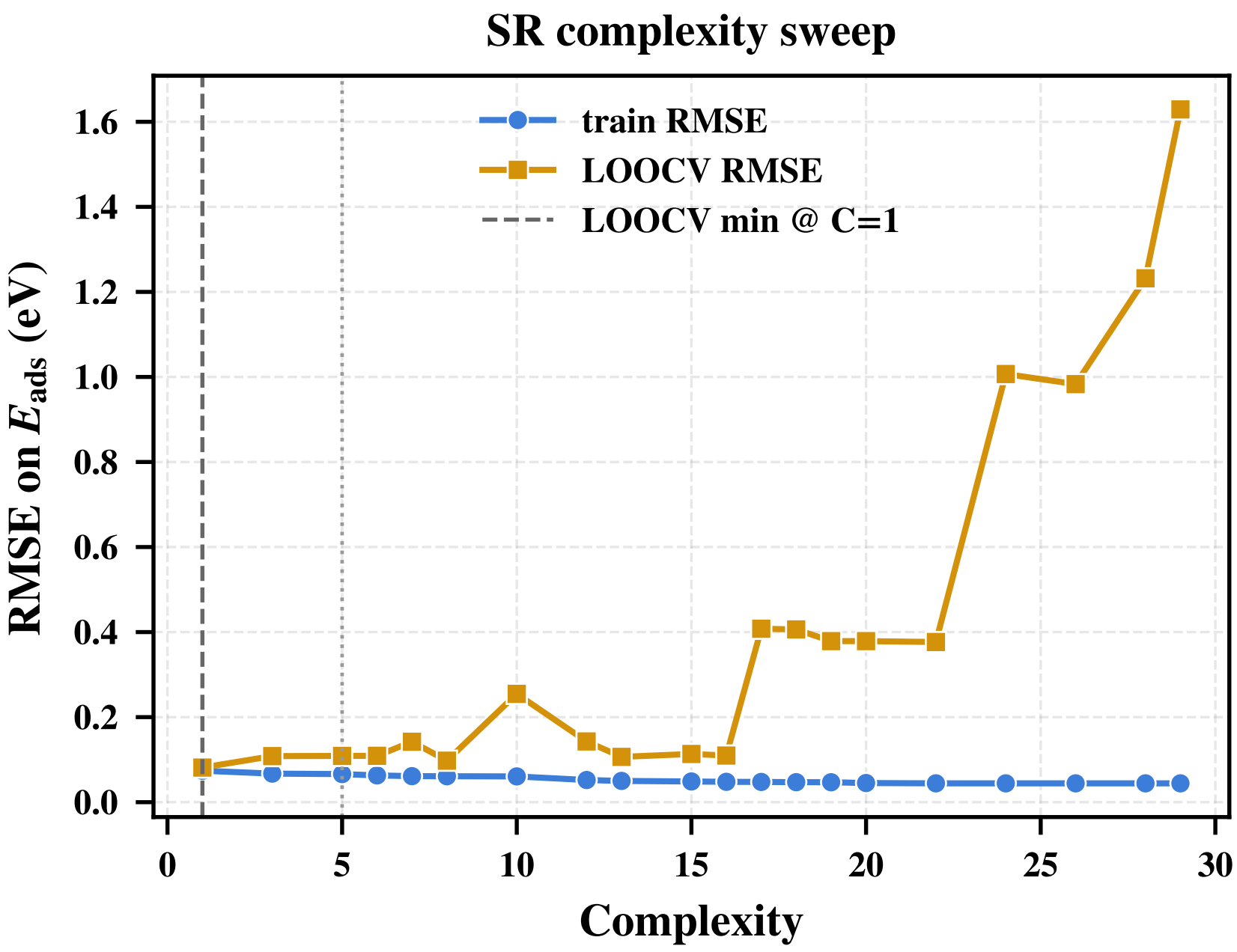

Figure S3: Symbolic-regression complexity sweep at $n = 11$. Train RMSE (blue circles) and LOOCV RMSE (orange squares) versus expression complexity. The dashed vertical line marks the LOOCV minimum, and the dotted vertical line marks the $C = 5$ complexity discussed in the main text. Training error decreases smoothly with complexity, while out-of-fold error shows a sharp overfitting cliff at $C \approx 17$. The high-complexity expression ($C = 28$, the highest non-dominated point on the Pareto front) therefore falls deep inside the overfitting regime, motivating its demotion to Figure S1 and the promotion of the low-complexity ω^--only expression at $C = 5$ as the LOOCV-stable interpretable model.

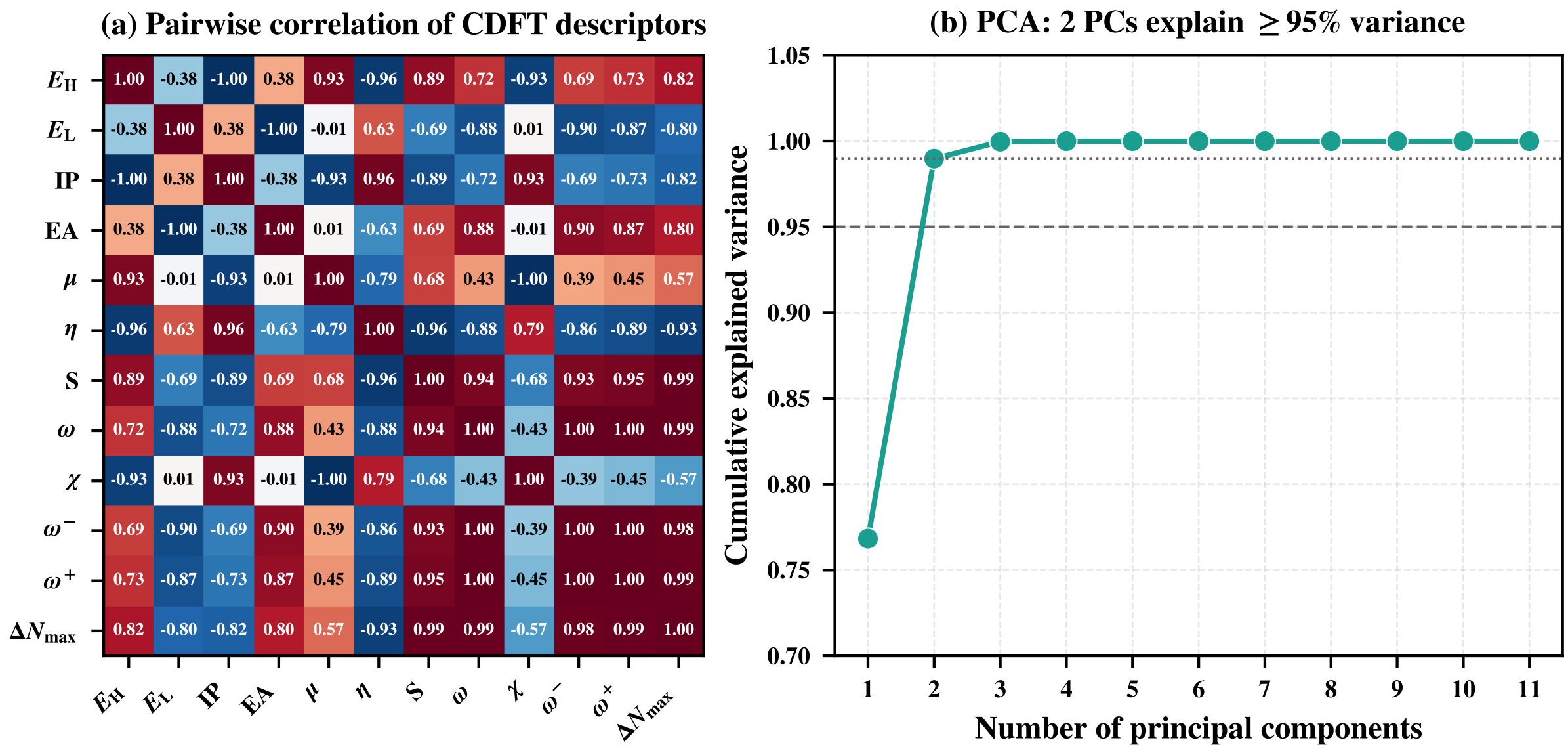

Figure S4: Descriptor-set correlation structure. (a) Pairwise Pearson correlation matrix of the twelve CDFT descriptors over the $n = 11$ doped systems used in the ML analyses. (b) Cumulative explained variance from principal-component analysis on the standardized descriptor matrix; horizontal lines mark the 95% and 99% thresholds. Two principal components explain more than 95% of the descriptor variance and three components explain more than 99%, indicating that the effective descriptor dimensionality is far smaller than the nominal count of twelve.

hofer, P.; Weiss, R.; Dubourg, V.; Vanderplas, J.; Passos, A.; Cournapeau, D.; Brucher, M.; Perrot, M.; Duchesnay, E. Scikit-learn: Machine Learning in Python. *J. Mach. Learn. Res.* **2011**, *12*, 2825–2830.

Rasmussen, C. E.; Williams, C. K. I. *Gaussian Processes for Machine Learning*; MIT Press: Cambridge, MA, 2006.

Settles, B. *Active Learning Literature Survey*; Computer Sciences Technical Report 1648; University of Wisconsin–Madison, 2009.
